# Design, implementation and usability analysis of patient empowerment in ADLIFE project via patient reported outcome measures and shared decision making

**DOI:** 10.1186/s12911-024-02588-y

**Published:** 2024-06-28

**Authors:** Gokce B. Laleci Erturkmen, Natassia Kamilla Juul, Irati Erreguerena Redondo, Ana Ortega Gil, Dolores Verdoy Berastegui, Esteban de Manuel, Mustafa Yuksel, Bunyamin Sarigul, Gokhan Yilmaz, Sarah N. L. I. M. Choi Keung, Theodoros N. Arvanitis, Thea Damkjaer Syse, Janika Bloemeke-Cammin, Rachelle Kaye, Anne Dichmann Sorknæs, on behalf of the ADLIFE study group

**Affiliations:** 1 SRDC Software Research Development & Consultancy Corp, ODTU Teknokent Silikon Blok Kat, 1 No:16 Cankaya, Ankara, 06800 Turkey; 2 Medical & Emergency Department M/FAM, OUH, Svendborg Hospital, Baagøes Allé 15, 5700 Svendborg, Denmark; 3 Biosistemak Institute for Health Systems Research, Torre del Bilbao Exhibition Centre, Ronda de Azkue 1, 48902 Barakaldo, Basque Country, Spain; 4 Electrical and Systems Engineering, School of Engineering, University of Birmingham, Birmingham, B15 2TT UK; 5 OptiMedis AG, Burchardstrasse 17, 20095 Hamburg, Germany; 6 Samson Assuta Ashdod Hospital, Ha-Refu’a St 7, 7747629 Ashdod, Israel; 7 TNO, The Netherlands Organization for Applied Scientific Research, Hague, Netherlands; 8 Odense University Hospital, Odense, Denmark

**Keywords:** Chronic Disease Management, Patient Reported Outcome Measures, Shared Decision Making, Decision Aids, Interoperability, Patient Empowerment Platform

## Abstract

**Introduction:**

This paper outlines the design, implementation, and usability study results of the patient empowerment process for chronic disease management, using Patient Reported Outcome Measurements and Shared Decision-Making Processes.

**Background:**

The ADLIFE project aims to develop innovative, digital health solutions to support personalized, integrated care for patients with severe long-term conditions such as Chronic Obstructive Pulmonary Disease, and/or Chronic Heart Failure. Successful long-term management of patients with chronic conditions requires active patient self-management and a proactive involvement of patients in their healthcare and treatment. This calls for a patient-provider partnership within an integrated system of collaborative care, supporting self-management, shared-decision making, collection of patient reported outcome measures, education, and follow-up.

**Methods:**

ADLIFE follows an outcome-based and patient-centered approach where PROMs represent an especially valuable tool to evaluate the outcomes of the care delivered. We have selected 11 standardized PROMs for evaluating the most recent patients’ clinical context, enabling the decision-making process, and personalized care planning. The ADLIFE project implements the "SHARE approach’ for enabling shared decision-making via two digital platforms for healthcare professionals and patients. We have successfully integrated PROMs and shared decision-making processes into our digital toolbox, based on an international interoperability standard, namely HL7 FHIR. A usability study was conducted with 3 clinical sites with 20 users in total to gather feedback and to subsequently prioritize updates to the ADLIFE toolbox.

**Results:**

User satisfaction is measured in the QUIS7 questionnaire on a 9-point scale in the following aspects: overall reaction, screen, terminology and tool feedback, learning, multimedia, training material and system capabilities. With all the average scores above 6 in all categories, most respondents have a positive reaction to the ADLIFE PEP platform and find it easy to use. We have identified shortcomings and have prioritized updates to the platform before clinical pilot studies are initiated.

**Conclusions:**

Having finalized design, implementation, and pre-deployment usability studies, and updated the tool based on further feedback, our patient empowerment mechanisms enabled via PROMs and shared decision-making processes are ready to be piloted in clinal settings. Clinical studies will be conducted based at six healthcare settings across Spain, UK, Germany, Denmark, and Israel.

**Supplementary Information:**

The online version contains supplementary material available at 10.1186/s12911-024-02588-y.

## Background

Chronic diseases are the leading cause of death and disability worldwide, accounting for two thirds of the global burden of disease and imposing significant healthcare costs [1]. Chronic illnesses present patients with significant challenges too: enduring conditions call for ongoing and intricate care, with disease and treatment demands evolving over time, demanding continuous decision-making and adaptations from the patient's perspective as well.

In response to these challenges, patient empowerment has gained attention in chronic disease management, to facilitate patient independence, self-management, and self-efficacy, by increasing patients’ knowledge about their health condition, and enabling them to participate in healthcare decisions [2]. As more care and treatment is carried out at home, patients and their caregivers need to be trained in making decisions regarding their lifestyle and illness in collaboration with healthcare professionals. This requires patients to have the necessary knowledge, abilities, and motivation to face these challenges [3, 4]. Successful long-term management of patients with chronic conditions requires active patient self-management and a proactive involvement of patients in their healthcare and treatment [5]. This calls for a patient-provider partnership within an integrated system of collaborative care, including self-management, education, follow-up and shared decision making [6].

One of the key instruments for empowering patients include using Patient Reported Outcome Measurements (PROMs) as tools for capturing the patient’s perspective on the outcomes of their own treatment and care [7]. PROMs are questionnaires completed by patients to ascertain perceptions of their health status, level of impairment, disability, and health-related quality of life [8]. They allow the measurement of outcomes in relation to clinical interventions from the patients’ perspective and represent a means of assessing clinical effectiveness and safety [9, 10].

Another important tool for patient empowerment is enabling Shared Decision-Making (SDM). There are different ways of defining Shared Decision-Making (SDM). The definition chosen for this research study is by one of the founders of the SDM theory; Glyn Elwyn. He defines SDM as: “An approach where clinicians and patients share the best available evidence when faced with the task of making decisions” [11]. As a new way to change the role of patients and their relationship with medical practitioners, SDM encourages the cooperation between the two actors when a decision needs to be made [12]. The clinician is the expert on the disease-specific knowledge, and informs the patient about treatment options, risks, pros and cons. Following this, the patient as the expert on his/her own life, tells the clinician about lifestyle, experiences with the disease, preferences, and priorities.

SDM is particularly important for chronic diseases that often require long-term and potentially complicated or intensive treatments. For patients with chronic conditions, SDM is expected to result in improved self-management using the term in a broad sense; that is, not only management of prescriptions but also factors such as diet, exercise, self-monitoring and participation in self-management education courses [13].

SDM is inextricably linked to the use of Decision Aids, and most of the literature found describes SDM relating to evidence for the use of decision aids [14]. The results show that SDM interventions enabled via decision-aids are complex, but most of them had a positive effect improving: adherence, knowledge, decision quality and chronic illness care, reduced decisional conflict and decision self-efficacy, perceived health status, perceived symptom severity and have an economic benefit [12, 15–17].

This paper outlines the design, implementation, and initial usability results of the patient empowerment processes for patients with severe long-term conditions such as Chronic Obstructive Pulmonary Disease (COPD), and/or Chronic Heart Failure (CHF) in the ADLIFE project supported by European Union’s Horizon 2020 research and innovation programme (grant agreement No 875209) [18]. The detailed processes for the selection, design, and technical implementation of two key patient empowerment tools, namely Patient Reported Outcome Measures and Shared Decision-Making Processes, are elaborated. Results of the usability study are presented, describing how the collected feedback is utilized to update the patient empowerment tools, as a preparation to large-scale clinical validation study planned.

## Methods

The ADLIFE project aims to develop innovative, digital health solutions to support healthcare planning and provide personalized, integrated care for patients over 55 years old, with severe long-term conditions such as COPD, and/or CHF. As a part of this integrated care solution, ADLIFE delivers a patient empowerment platform supporting Patient Reported Outcome Measures (PROMs) and Shared Decision-Making (SDM) to support the patients in their daily lives for the management of their chronic conditions. The project will use and evaluate these technology innovations in six healthcare environments across Spain, UK (two sites), Germany, Denmark, and Israel as a part of large-scale clinical pilot study that will be completed in 2024 [19].

In this section, we first present the methods used in the design of the patient empowerment tools, namely PROMs and SDM interventions in the ADLIFE project to empower patients suffering from COPD and CHF to take an active role in the management of their diseases in cooperation with their healthcare professionals. Secondly, we describe in detail the methods we have used to implement these two mechanisms via the digital platforms served to healthcare professionals and patients. Finally, the design of the initial usability study conducted to gather user feedback to prioritize updates to the ADLIFE platform before the final clinical pilot study is presented.

### Selected PROMs for ADLIFE study

ADLIFE follows an outcome-based and patient-centered approach, where the effects of digital solutions will be evaluated to assess the impact to the health status and the quality of life of chronic disease patients (See Table 1). ADLIFE Project has chosen to use the International Consortium for Health Outcome Measures (ICHOM) standard set for older person [20] to define patient centered health outcomes. Following ICHOM terminology, we have selected health outcome areas (such as autonomy, functioning quality of life, clinical status) and dimensions for each of the selected area (such as symptom control, mood and emotional health) that we are targeting to assess, as listed in Table 1.

**Table 1:** The list of PROMs to be used in the ADLIFE project

ADLIFE areas	ADLIFE dimensions	PROMs
**Symptoms, functioning quality of life**	Autonomy, control	EQ-5D-5L [23]
	Symptom control	EQ-5D-5L [23]
	Mood and emotional health	EQ-5D-5L [23]
		HADS [33]
	Social context	EQ-5D-5L [23]
	Activities of daily living	EQ-5D-5L [23]
		Lawton IADL [31]
		Barthel Index [32]
**Clinical status**	Complexity (i.e. hurdle, severity)	CAT [24]
		mMRC [25]
		KCCQ [30]
**Healthcare responsiveness**	Participation	Shared decision making: “ask 3 questions” [26]
**Care**	Carer burden	ZBI-22 [28]
		WEMWBS [29]
	Satisfaction	PCQ-P [27]

PROMs represent an especially valuable tool to evaluate the outcomes addressed in this project as a part of clinical pilot study. PROMs enable the measurement of outcomes in relation to clinical interventions from the patients’ perspective and represent a means of assessing clinical effectiveness and safety [9, 10]. These questionnaires are completed by patients to ascertain perceptions of their health status, level of impairment, disability and health-related quality of life [7, 21]. Selected PROMs for ADLIFE will allow evaluating the most recent patients’ clinical context, constituting a supportive tool for health status assessment, the decision-making process, and the definition of care goals and activities according to the patients’ specific needs.

It is important to use valid, reliable, and appropriate instruments when selecting PROMs and minimize the burden on patients and healthcare teams in data collection. Depending on the target, PROMs can be generic, disease-specific, or condition-specific [7]. The advantage of generic PROMs is that they allow comparison of outcomes across conditions [22]. There are also a large number of disease-specific PROMs. When used together, generic and disease-specific PROMs can provide complementary information [7].

The definition of the specific PROMs relevant for ADLIFE (i.e. PROMs that will be useful to measure the health outcomes described in the ADLIFE clinical pilot study [19]) has been a crucial step of this project. The process has been conducted by the working teams created in pilot sites participating in ADLIFE project, (the Basque Country (Osakidetza), United Kingdom (NHSL Lanarkshire), Poland (FALKHOSP Lower Silesia), Denmark (Southern Denmark), Germany (Werra-Meißner Kreis), RJH-Sweden (Region Jämtland Härjedalen) and Israel (Assuta Ashdod Hospital and Maccabi Healthcare Services). These teams are comprised of members of a multidisciplinary group of health professionals (hereinafter referred to as the ‘Clinical Reference Group’ (CRG)) such as General Practitioners, nurses and specialists. RJH-Sweden has later decided to not to carry out pilot studies with patients and care givers, however they have continued to contribute to CRG, sharing their expertise in integrated care processes. On a later stage, pilot from United Kingdom (UHCW, University Hospitals Coventry and Warwickshire – NHS Trust) joined the project as a new pilot site replacing Polish pilot and it has accepted to implement the agreed final PROMs list.

CRG has contributed to defining the PROMs that should be collected to provide useful information to assist in the evaluation of the patients’ health status and the clinical decision-making process. After a detailed research for the most suitable tools to measure the health outcomes addressed in ADLIFE, CRG has agreed to include a list of PROMS (Table 1) matching the ADLIFE health-related areas including: symptoms, functioning quality of life, clinical status, healthcare responsiveness and care. In this process, CRG has evaluated the PROMS in terms of adequacy and coherence with the project in terms of the intended use, relevance, and feasibility for collecting and retrieving them. An additional criterion considered in the selection of PROMs was whether the questionnaires were available in the different languages spoken in each of the pilot sites (a total of 7 different languages, English, Danish, German, Hebrew (Israel), Russian (Israel) and Spanish). All of the selected PROMs included in the final set list are available originally in English version and available in most of the languages which patients are expected to speak in each pilot site. Where necessary, translations to additional languages are carried out and validated as well based on the translation and linguistic validation guidelines provided by the publishing organizations of the respective questionnaires (e.g. MAPITrust for EQ-5D-5L and WEMWBS).

The final list of PROMs implemented and used in ADLIFE is the following (see Table 1): (i) The 5-level EQ-5D version (EQ-5D-5L) [23]; (ii) The COPD Assessment Test (CAT) [24]; (iii) The Modified Medical Research Council Dyspnea Scale (mMRC) [25]; (iv) The Shared decision-making: “ask 3 questions” [26]; (v) The Person-centered Climate Questionnaire – patient version (PCQ-P) [27]; (vi) The Zarit Burden Interview: 22-item version (ZBI-22) [28]; (vii) Wellbeing questionnaire (WEMWBS) [29]; (viii) Kansas City Cardiomyopathy Questionnaire (KCCQ) [30], (ix) Lawton Instrumental Activities of Daily Living Scale (IADL) [31], (x) Barthel Index [32], and (xi) Hospital Anxiety and Depression Scale (HADS) [33].

### Shared decision making and decision aids in ADLIFE

The care model suggested in ADLIFE will facilitate a more active role of patients and caregivers in their own health and symptom management by implementing shared decision-making (SDM) and offering individualized adaptive interventions. This patient-centered approach, in which the patients’ values and preferences are incorporated, enable the definition of an individualized and personalized treatment for patients.

Despite professionals indicating that they consider it important to share decisions with patients [34], SDM seems to be applied in daily practice to a limited extent only. The primary barrier to the adoption of SDM in practice is clinical perception that SDM is not pertinent to the decisions they are making with patients [35]. In addition, implementation of SDM into daily clinical practice may seem inapplicable in the busy and highly responsible work of a doctor: the dilemma about the time consumption of the conversation and the impact on the clinical decisions [36]. As SDM cannot be successfully implemented without the goodwill of the clinicians [37], the ADLIFE projects promotes, encourages and offers the possibility of promptly integrating SDM through its digital integrated care platforms for the use of healthcare professionals and patients.

The ADLIFE integrated care solution provides two complementary software platforms for the use of healthcare professionals and patients: (1) A Personalized Care Plan Management Platform (PCPMP) supported by clinical decision support services, which acts as a chronic disease management platform served to multidisciplinary care team members (specifically GPs and Nurses), and (2) A Patient Empowerment Platform (PEP) used by the patients and their informal care givers, enabling them to be informed, educated, and guided about their active care plan and to be active participants of their care plan activities.

The PCPMP serves the multi-disciplinary care team members and facilitates the creation of personalized care plans for patients. It retrieves important parameters from the Electronic Health Records (EHR), and invokes Clinical Decision Support Services (CDSS), to recommend personalized suggestions about care plan goals and activities. The suggestions of the CDSS are produced by automatized evidence-based clinical guidelines, that support healthcare professionals in creating a care plan for the patient.

As part of the care plan, roles and responsibilities of the patient in the management of his chronic condition are clearly defined. Once the care plan is finalized, this care plan is then shared with the multidisciplinary care team members via PCPMP, and with the patient and his/her informal caregivers via PEP. In this way, the care plan and all its components can be accessed by the patients. PCPMP and PEP also enable shared decision-making while the personalized care plan is being created based on the preferences of the patients following a patient-centered approach.

**Fig. 1:**
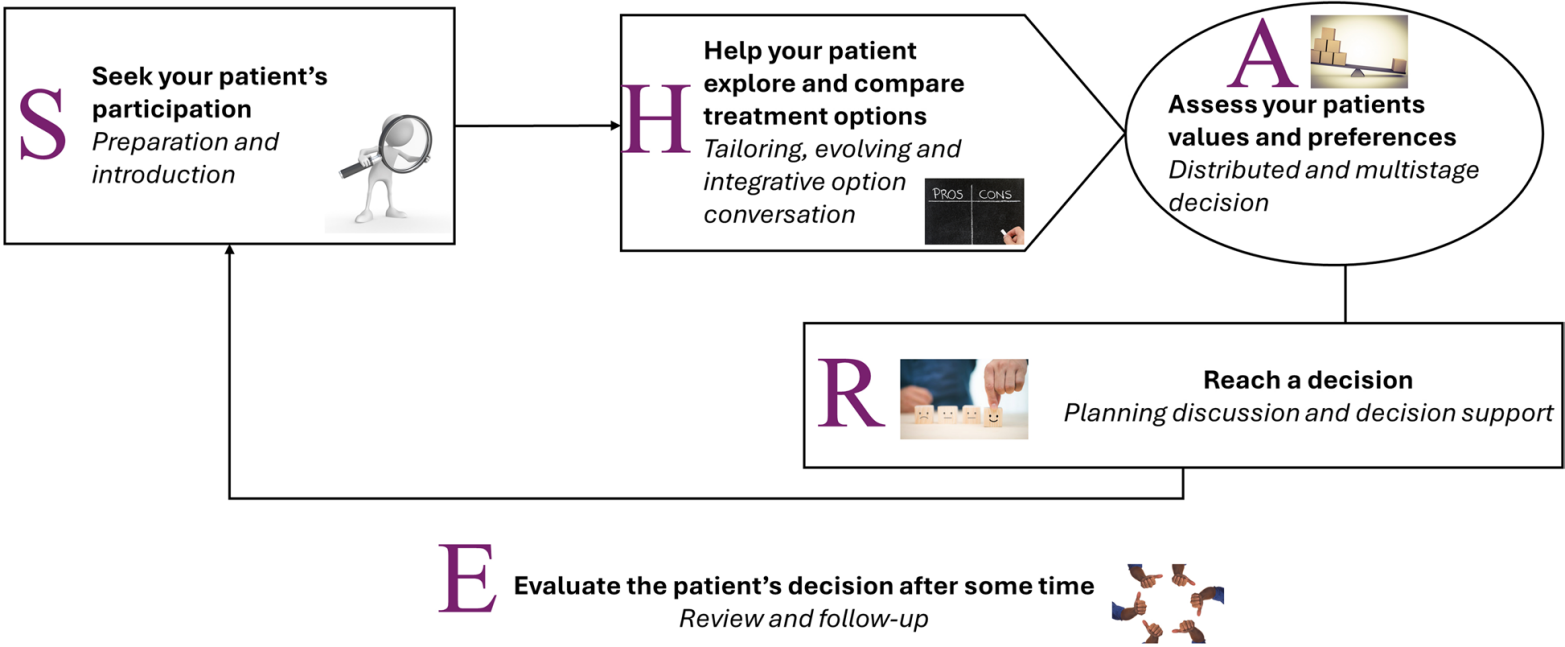
The SHARE Approach implemented in ADLIFE

From at least 22 different approaches of SDM that exist [38], the ADLIFE project implements the "SHARE approach [39]”, a generalized SDM model that streamlines the nine essential steps of SDM identified by Makoul and Clayman, into five tasks: (1) Seek the involvement of your patient (practitioner), (2) Helping the patient explore and compare treatment options, (3) Assess the patient’s values and preferences, (4) Make a decision, (5) Evaluating the patient’s decision after a period of time (Fig. 1).

The SHARE approach proposes the involvement of patients and professionals in different tasks such as information transfer, risk communication and preferences elicitation, tailoring options or broader decision making. The five steps model used in ADLIFE included assets and information related to implementation processes, the identification of activities to be carried out in each task of S–H-A-R-E approach (Fig. 1), skills that professionals and patients should acquire for applying SDM activities, and factors that influence the process and potential 'decision aids' enabling SDM.

The SHARE workflow will start when a decision needs to be reached as part of the patient’s care plan in ADLIFE. Those situations have been identified in the NICE evidence-based clinical guidelines for COPD, Heart Failure, and other comorbidities by the ADLIFE CRG. Fact sheets with task definition, the triggers of the task, the aids for professionals, the aids for patients and the scenario in which aids are offered in specific situations are identified based on the evidence-based clinical guidelines utilized in ADLIFE. In Table 2, we summarize the information provided to the CRG as a template for designing each task of the S–H-A-R-E approach and identifying shared decision-making processes that can be provided via the ADLIFE toolbox.

**Table 2:** Implementation of SHARE approach in ADLIFE

	**TASK DEFINITION**	**TRIGGERS**	**AIDs FOR PROFESSIONALS**	**AIDs FOR PATIENTS**	**SCENARIO IN WHICH AIDS ARE USED**
Seek your patient's (professional’s) participation	Preparation: The professional seeks and starts engaging the patient before introducing the choices Introduction: The professional confirms with the patient that there is a choice, and we provide a rationale for choice	Selected decision points in the flowchart of the clinical guidelines or specific goals/activities By request (patient, caregiver, or professional)	Info cards that can introduce supporting tools via PCPMP: • Adapt the information and treatment options to the patients’ emotional states and circumstances. Consider the patient’s lifestyle and a way of life to perform the choice • Long-term chronic patient may be reluctant to actively manage that condition and less likely to engage in the SDM discussion • Anticipate patient doubts and the demand of information • There are different levels of patient participation possible; from fully involved to not involve at all	• Option to start the activity of SDM via a selected decision aid to be completed by the patient via PEP	• Preparation when reviewing the patient’s record and preparing the encounter • Introduction in an appointment via PCPMP
Help your patient explore and compare options	This iterative process aims to determine which of the alternative responses best reflects the patient’s preferences after personal reflection and feeling confident for making an informed decision Tailor, evolve and integrate an option conversation with the patient through presenting initial options, preference elicitation; tailoring options; checking understanding the options presented	After the S task Selected decision points in the flowchart of the clinical guidelines or specific goals/activities	Info cards that can introduce supporting tools via PCPMP: • Patients’ previous knowledge about the condition or available treatment options can be an influence. It is preferable to review treatment “facts” options with the patient • Social and cultural circumstances can influence in the options presented • Tailor the presentation of automatic proposed options to the patient and ensure that they are understood and address any knowledge error or gap • Keep the conversation active and plan next steps based on the results of the patient’s decision-making needs	• Educational materials available in the PEP will be used to help to better understand the condition	In an appointment: • virtually • face-to-face
Assess your patients’ values and preferences	Patient view of the options and values are discussed Characterized by multiple and multi-stage decisions, which are distributed among the patient, clinicians and sometimes other services	After the H task **Selected decision points** in the flowchart of the clinical guidelines or specific goals/activities	Info cards that can introduce supporting tools: • Perceived urgency to make a decision may influence on the SDM process, specifically it reduces the patient’s level of engagement • Review preferences in order to understand what “most matter” to the patient • Ensure and check there are no further and negatively influence decision-making needs • Based on patient vales and preferences rate the benefits and risks identified for each of the options listed	• Tools to carry out the activity (such as reviewing a selected decision-aid) can be offered in the PEP • Share the resulting rates with the professional prior consultation	• Patients’ home via PEP
Reach a decision	Consolidate or summarize preferences and moving towards a decision or an ongoing reflective and iterative process until the point a decision needs to be made Assess the patient's readiness to make a decision Negotiation with the patient a mutually agreed upon a course action	After the A task Selected decision points in the flowchart of the clinical guidelines or specific goals/activities	Info cards that can introduce supporting tools: • Give “informed preferences” about what might be suitable for the patient. Recommendations should be based on the information that the patient had given about their preferences and checking always the patient's understanding and agreement • Ensure that the decision is taken based always in patient’s life style and preference • If applicable and necessary, check on a review frequency	• Confirm the decision via PEP • Make a summary of the next steps. (if applicable.)	• PCPMP • PEP • In an appointment: virtually or face-to-face
Evaluate the patient's decision after some time	Review and revisit the decisions reach by the patient, if the decision aid has been used, whether a problem occurred and others Discuss follow-up	Set in the review frequency	Info cards that can introduce supporting tools: • All decision aids are not created equal. Revise tools to help you evaluate the quality and usability of patient decision aids and other evidence-based resources • Follow-up patient and evaluate the treatment, care or choice taken. A good option is to set a reminder to carry out this activity	• Reminder for reviewing decision	In an appointment: • virtually • face-to-face

**Fig. 2:**
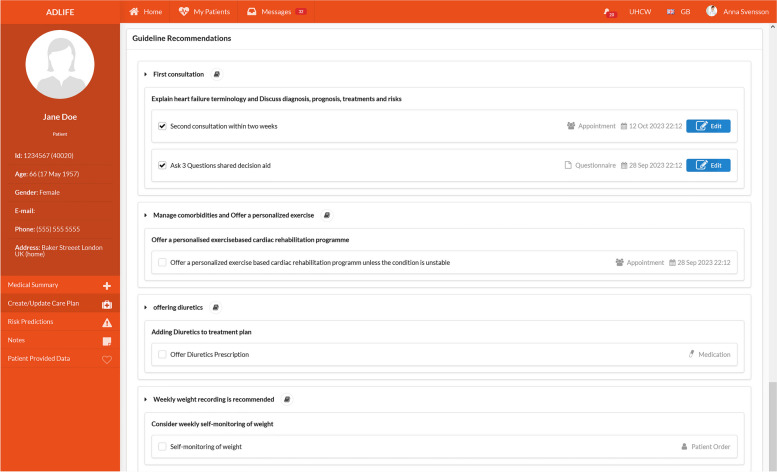
ADLIFE PCPMP interface suggesting the ‘Ask 3 questions decision aid’ to be assigned to the patient

To implement shared decision-making directly into the ADLIFE platforms, trigger points from evidence-based clinical guidelines were reviewed by the CRG to identify an opportunity and the potential task for utilizing the SHARE model between the patient and the clinician. More than 40 trigger points were identified in the clinical flowcharts with slightly more than half pertaining to medication options. The shared decision-making trigger points are eminently suited for integration into the PCPMP and PEP platforms. Each trigger point has been turned into a prompt on the PCPMP through the application of the CDSS (Clinical Decision Support Service) framework, where the progression through the clinical flowcharts triggers a pop-up notification stating that there is an opportunity for initiating the SHARE model. Here, healthcare professionals are provided with information as depicted in Table 2, as information cards. In Fig. 2, an example interface from PCPMP is presented where the clinician is suggested to add the ‘Ask 3 questions decision aid’ as a questionnaire to the patient. For some of the trigger points PCPMP suggests that healthcare professionals assign educational materials to the patient, that can be reviewed on PEP.

Finally, two well-structured decision aids have been selected (‘Ask three questions’, and ‘Shared decision-making on inhalation medicine in patients with COPD’) to be implemented. These are offered to healthcare professionals at the identified trigger points in the PCPMP, to be added to the care plan of the patient. Once they are added to the care plan and assigned to the patient, the patient can see, review, and complete them in PEP. The feedback from the patient is seen on the PCPMP by the healthcare professional. As an example, before an inhalation device is to be prescribed to the patient in the scope of a care plan for COPD patients, the healthcare professional can assign the ‘Shared decision-making on inhalation medicine in patients with COPD’ to the patient, to determine the most suitable inhalation medication for a patient by having them prioritize different factors such as minimizing the frequency of inhalation medication intake, minimizing the number of different inhalation devices used daily, and minimizing the cost of medication. The implementation of decision aid is detailed in Sect. 2.3.

There are too many local, cultural, and practical variations in all the possible answers to each of the steps in the SHARE model across the pilot sites to build bespoke prompts for all the trigger points. The clinical guidelines are likely to change over time and available treatment options may also change in the pilot sites during the intervention. These can be easily reflected in the PCPMP by updating the CDSS triggers, and the information cards to be presented to the healthcare professionals via PCPMP.

### Technical implementation of PROMs and decision aids in ADLIFE architecture

As part of the care plan management, an important feature enabled via the digital ADLIFE Toolbox (PEP and PCPMP) is the collection of feedback from the patient via PROMs and via symptom reporting questionnaires and also enable shared decision making about the care plan activities. ADLIFE PEP and PCPMP are built upon international standards, and interoperability of data exchange among these components is enabled via HL7 Fast Healthcare Interoperability Resources (FHIR) standard [40]. We are using an HL7 FHIR Repository as the common data repository that enables seamless data exchange between local EHRs, PCPMP and the PEP. In our architecture we are using the open source on FHIR.io FHIR Repository [41].

In the following sections we will be focusing on presenting the details of the implementation of PROMs, symptom reporting questionnaires, shared decision-making processes and decision aids in the ADLIFE architecture. The overall details of ADLIFE PEP platform are presented in our recent article [42].

#### Implementation of PROMs in ADLIFE architecture

In the ADLIFE architecture, we have followed the HL7 FHIR Patient Reported Outcomes Implementation Guide [43] to represent PROMs in a machine processable manner following an international standard. PROMs are represented as HL7 FHIR Questionnaire Resources, in this way they can be stored, exchanged between different health IT systems, and processed seamlessly addressing the interoperability challenge. An example HL7 FHIR Questionnaire Resource representing Kansas City Cardiomyopathy Questionnaire (KCCQ) is presented in Additional file 1. These are included into the care plan of the patient by the physicians creating the care plan via PCPMP. PCPMP with the help of CDSSs can trigger this. They are represented as Service Requests in the care plan as activities to be carried out by patients periodically. These Service Requests are then parsed by the PEP tool, to present the PROMs to be filled in by patients clearly. An example of such an assignment is presented within a care plan resource in FHIR format presented in Additional file 2. The Care plan resources defined in HL7 FHIR, including references to PROM questionnaire resources are created by PCPMP interfaces, and saved to the common data repository.

**Fig. 3:**
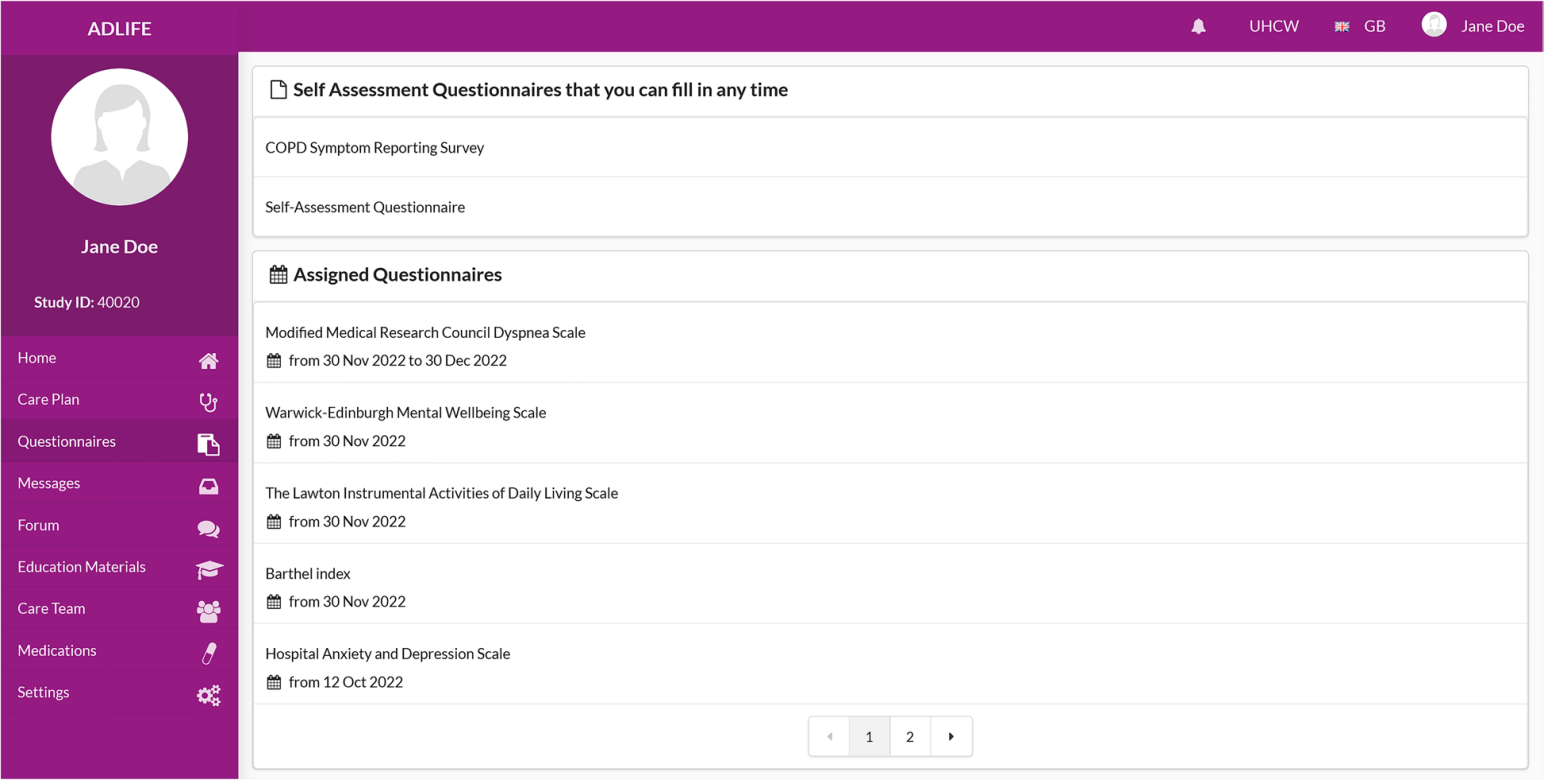
ADLIFE PEP Portal listing questionnaires assigned to the patient

The care plan resources are retrieved as a bundle by PEP whenever a new care plan is created or updated from the common data repository. After this, the PROM assignments are presented to the patient as part of their care plan and rendered as user-friendly web-based surveys, enabling the patient to easily fill them. An example snapshot from ADLIFE Web based PEP portal, listing the questionnaires assigned to the patient is presented in Fig. 3.

**Fig. 4:**
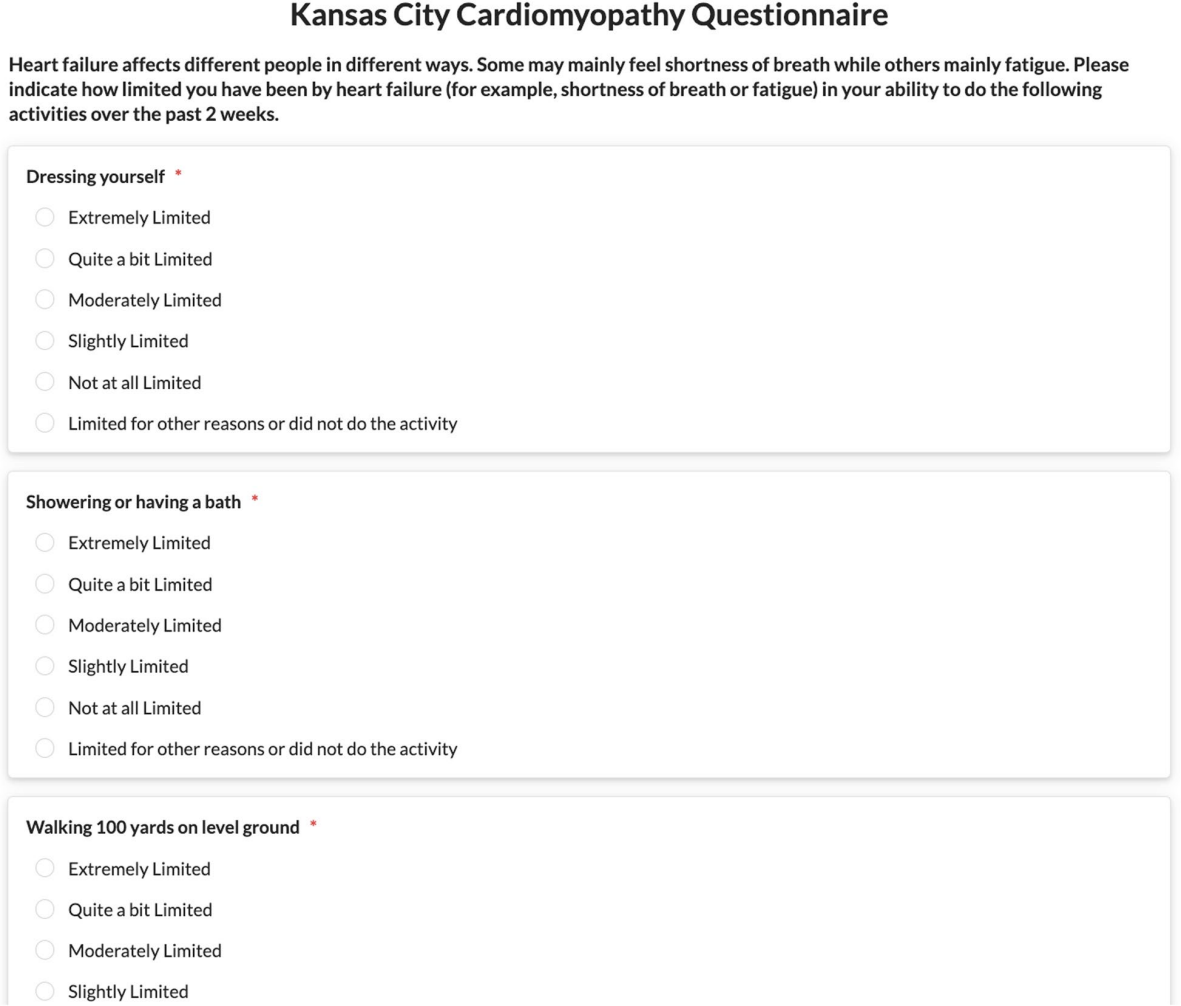
A Multiple-choice question from PEP Web Portal (KCCQ)

**Fig. 5:**
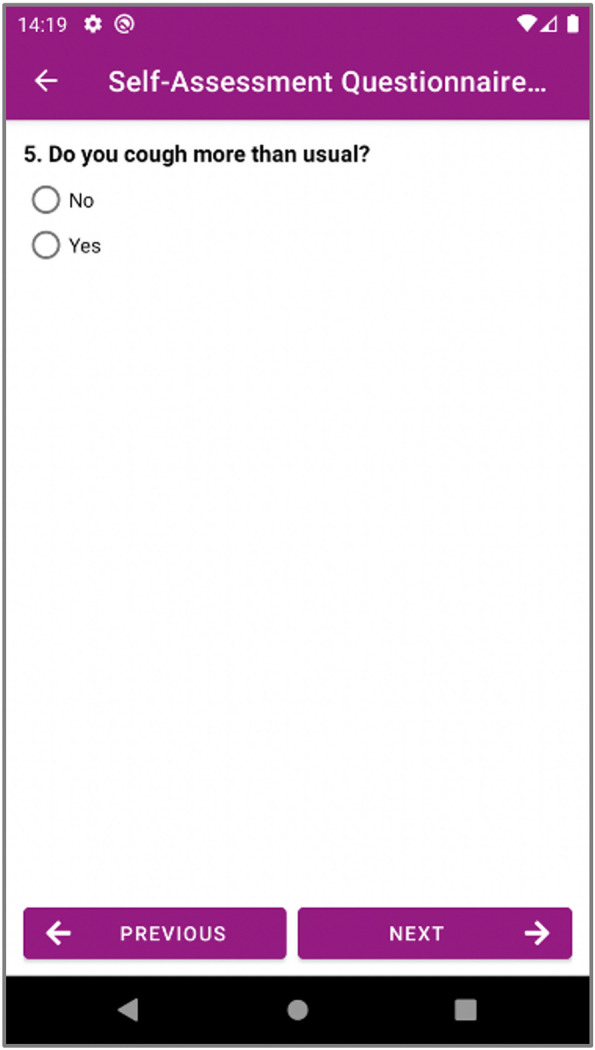
A Yes/No question from Mobile PEP

PEP interfaces have been built to automatically render these machine-processable questionnaire definitions to present them to the patients and enabling patients to fill in these questionnaires easily. Depending on the content of the questionnaire we have enabled different presenting views to collect the responses via multiple choices, via Yes/No questions, via a slider, or via free text. Different views from web-based PEP Portal and mobile PEP application are depicted in Figs. 4 and 5.

The responses are recorded as FHIR Questionnaire Response Resources and saved back to the FHIR Repository. An example KCCQ questionnaire response as a FHIR resource is presented in Additional file 3.

When the PROM includes a scored assessment, the resulting score is represented as an Observation Resource that is linked with the PROM. Once each PROM is completed in the PEP by the patient or the informal caregiver, the resulting questionnaire response is sent to a decision support service we have implemented to determine if there is a score attached with the PROM or not. The CDSS, triggered automatically by PEP, receives the questionnaire response, determines if it is a scored assessment, calculates a score if it is and creates an Observation resource to be put in the FHIR repository. The score observation contains a reference to the Questionnaire Response resource indicating that it is created as a result of that specific response instance. The observation might also contain an interpretation of the score, if it can be interpreted by PROM definition. Additional file 4 contains a resulting score Observation resource created from a KCCQ response.

**Fig. 6:**
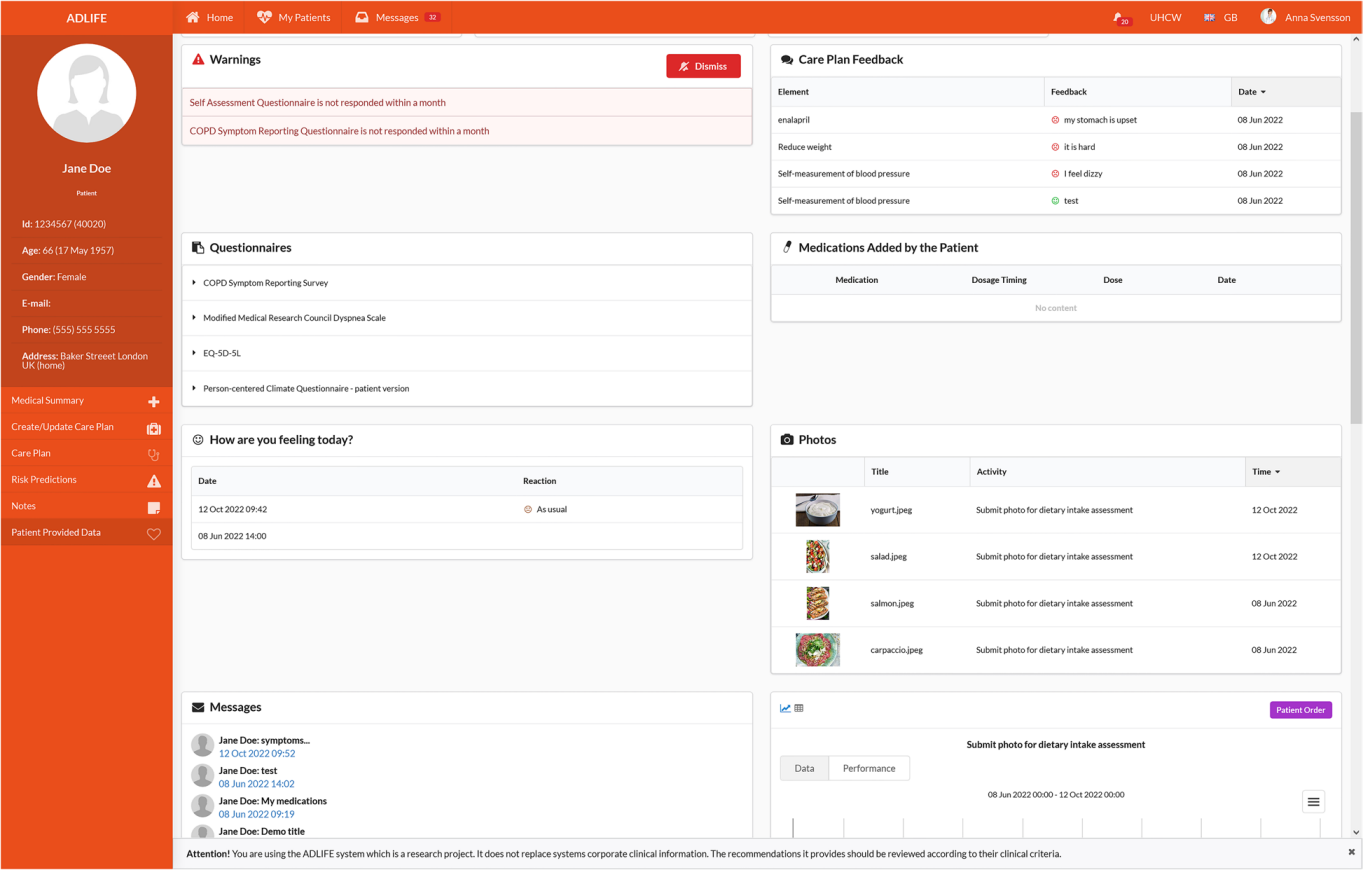
PCPMP Interfaces presenting the Questionnaires filled by the patient

The PCPMP, which is designed to receive notifications whenever a new PROM questionnaire is fulfilled, is informed, and the responses are made available to the practitioners as depicted in Fig. 6.

ADLIFE PEP aims to enable patients to report their symptoms as part of their responsibility in shared care plan management. The CRG has selected following symptoms listed in Table 3 are decided to be collected via PEP through the selected tools:

**Table 3:** List of symptoms to be collected via PEP

Conditions	Symptoms to be collected and Tools to be used
COPD	A specific adaptive questionnaire has been designed for COPD Symptom Reporting. This questionnaire is presented in Additional file 5. In addition to this, COPD Assessment Test (CAT) [24] will be used, which has already been implemented as a PROM
Generic (For all patients)	We have designed the Self-Assessment Questionnaire as a means to be complementary to the care plan and to collect information about the perceived change in symptoms in an easy and simple way. This questionnaire is presented in Additional file 6. It is already validated in CareWell European project [44] It can be seen as a set of self-checking questions to help the patient learning about the warning signs (self-control), and provide guidance about what actions need to be carried out, in case of symptoms (such as ‘You have perceived changes in your breathing and swollen legs, review your care plan and call your nurse or doctor for advice’). The responses are also saved and shared with healthcare professionals via PCPMP
Diabetes	A short questionnaire has been designed to ask for new gastrointestinal symptoms 2–3 weeks after initiation with metformin, and provide feedback to the patient via PEP
Mild Cognitive Impairment and Depression	Global Depression Scale (GDS), Hospital Anxiety and Depression Scale (HADS) and Montgomery and Asberg Depression Rating Scale (MADRS) has been decided to be used. The Physician will decide which one to use while s/he is preparing the care plan
Hepatopathy	Alcohol Screening Tool (AST) and Fast Alcohol Screening Tool (FAST) have been selected to be used to be assigned to patient via PEP
Hepatopathy	A short questionnaire has been designed to ask for ‘Nausea and itching skin’ as liver disease symptoms
Heart Failure	Modified Medical Research Council Dyspnea Scale (MMRC) has been decided to be used to record ‘decrease in physical functioning’

All these additional questionnaires have also been represented as FHIR resources and made available to PCPMP so that healthcare professionals can assign them to their patients as part of their care plan to be filled via PEP.

As a result of discussion in the CRG, it was decided to notify healthcare professionals via PCPMP as warnings when certain symptoms are reported via these questionnaires as follows.

- Once the patient fills in the COPD Symptom Reporting Questionnaire, the system automatically checks whether based on the flow (see Additional file 4), a worsening in symptoms is detected. In this case a specific HL7 FHIR Observation is created to represent this as a red flag.
- Similarly, the system checks the responses to the general self-assessment test (See Additional file 5), if any symptom is reported, a specific HL7 FHIR Observation is created to represent this as a red flag.
- For CHF patients, if swollen legs, or increased cough is reported in the general self-assessment test, a specific HL7 FHIR Observation is created to represent this as a red flag.
- For CHF patients, if there has been 1.5 kg change in the recorded weight in a period equal or less that one week, a specific HL7 FHIR Observation is created to represent this as a red flag.
- Finally, if the patients have not filled in COPD Symptom Reporting Questionnaire or general self-assessment test in the last month at all, the system detects this and a specific HL7 FHIR Observation is created to represent this as a red flag.

These automatically created red-flag observations are presented to the healthcare professionals as warnings in PCPMP as presented in Fig. 6.

#### Implementation of decision aids in ADLIFE architecture

The first decision aid implemented is the ‘Ask Three Questions [26]’. Research shows that encouraging patients to ask three simple questions that leads clinicians to provide higher-quality information about options and their benefits and harms. The three questions are: (1) What are my options? (2) What are the pros and cons of each option for me?; and (3) How do I get support to help me make a decision that is right for me? An optional fourth question may also be asked: ‘What if I do nothing?’.

Via the PEP, the patient is always able to raise these questions. In addition to these, we have enabled the patient to add 5 additional free text questions which will be delivered to the healthcare professional via PCPMP to be discussed during the next care plan review meeting.

The second tool is the ‘Shared decision-making on inhalation medicine in patients with COPD’. It is a tool that can be assigned to a patient, to assess which inhalation medication will best suit the patient, asking the patient to assess what is most important to them and what matters less. The patient is asked to prioritize different choices by assigning scores to each of them, while keeping the total score as 10 to indicate which option is more important for them. The options are as follows:

- Keeping the daily’frequency’ of inhalation medication intake (number of times you should take inhalation medication daily) as low as possible;
- Keeping the number of different inhalation devices you need to use daily as low as possible;
- Keeping the cost of medication as low as possible.

The complete description of this decision-making tool is available in Additional file 7. This tool is implemented in PEP as a questionnaire (see Additional file 8 for FHIR representation as a Questionnaire Resource). During the care planning session in PCPMP, the clinician can add this decision aid as an activity to be carried out by the patient via the PEP.

**Fig. 7:**
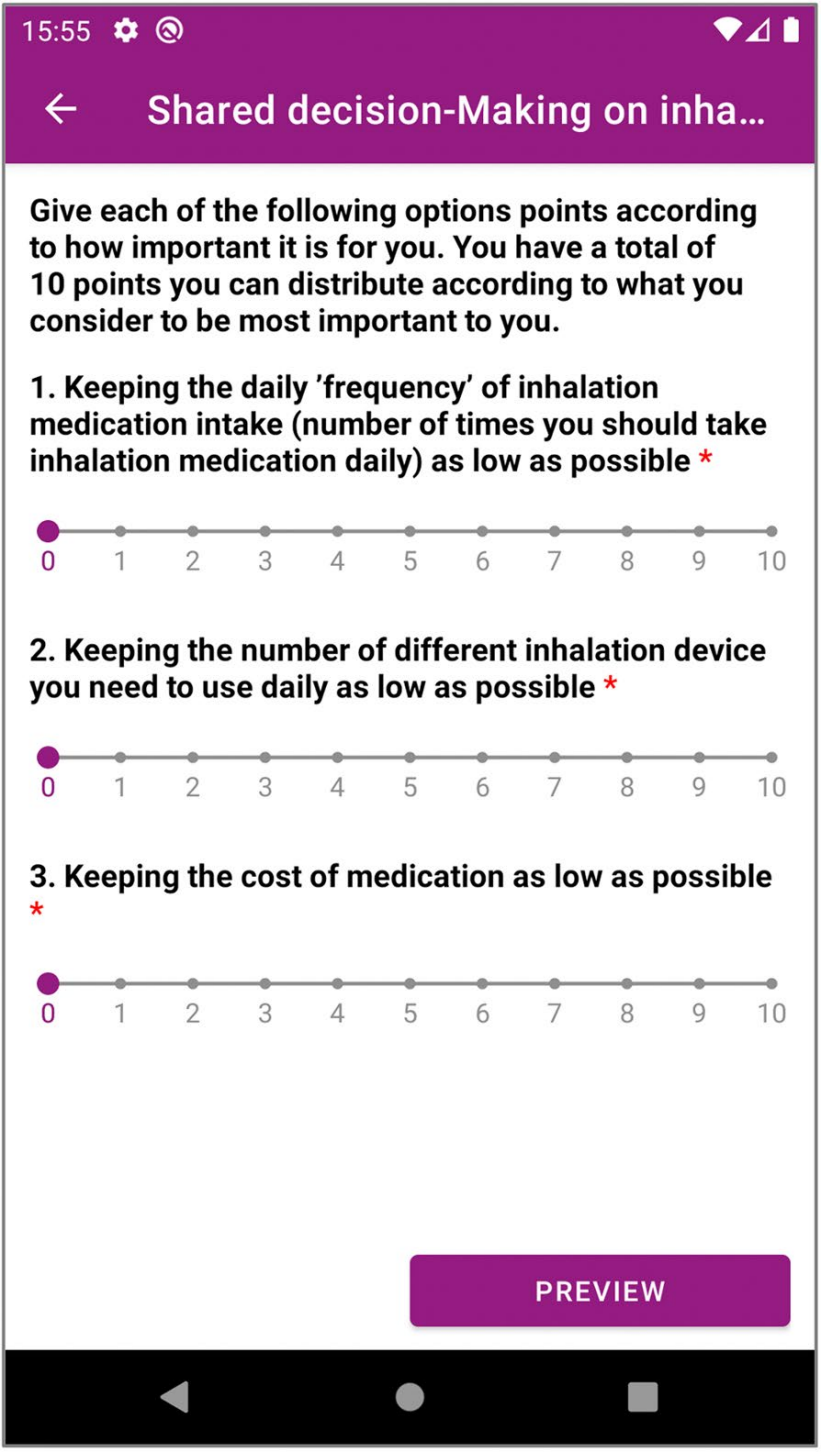
A snapshot from ‘Shared decision-making on inhalation medicine in patients with COPD’ tool from mobile PEP App

**Fig. 8:**
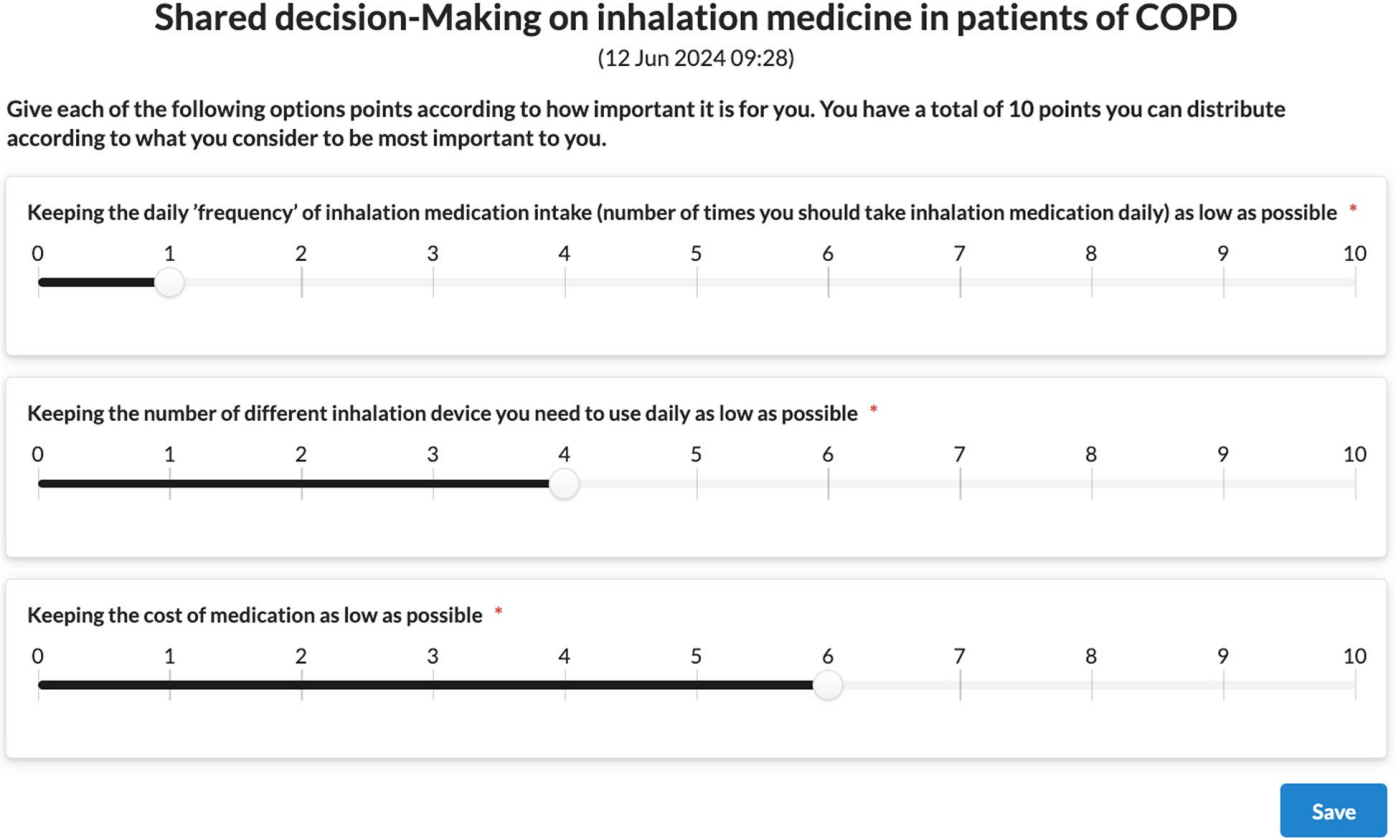
A snapshot from PCPMP presenting how health care professional sees the responses of the patient for the 'Shared decision-making on inhalation medicine in patients with COPD’ decision aid

This questionnaire is presented to the patient via PEP interfaces, and the user is guided in a step-by-step manner about how s/he should answer the questions. A snapshot from the mobile PEP App is presented in Fig. 7. The responses of the patient are shared with the healthcare professional via PCPMP as depicted in Fig. 8.

### Usability study design

The technical development of the ADLIFE PEP platform has been done in close collaboration with target end users, i.e., patients and healthcare professionals. This is to ensure that the requirements of end users are correctly elicited and taken into account and for any remaining issues to be resolved before the deployment of the platform for use during the ADLIFE clinical pilot study [19]. Between April and June 2022, a usability study was conducted to gather feedback and to subsequently prioritize updates to the ADLIFE toolbox before the clinical pilots are initiated at each site for the clinical pilot study. In this section, we detail the design of this usability evaluation study for the PEP platform.

At the time of the usability study, 2 sites had received local ethical and organizational approvals for recruiting patients and their informal caregivers for the study (Germany, Spain). These sites aimed to recruit between 3 and 5 participants each. The recruitment process is achieved from the same pool of patients that are eligible to be involved in the clinical pilot study planned. The participants attended a training workshop, where the PEP platform was demonstrated. Login credentials were provided to the participants to enable them to test the platform for themselves, using a typical user scenario. The participants were then asked to complete an online questionnaire to record their level of satisfaction with different aspects of the PEP platform. One site in the UK (England) has opted to collect feedback via a Patient and Public Involvement (PPI) approach [45] due to the timeline of the activity and the participants did not test the tool directly.

The standardized Questionnaire for User Interface Satisfaction v7 (QUIS7) [46, 47] was used to collect participant feedback in terms of participants’ opinions on usability and attitudes of acceptance for the system, with Likert scales for opinions and free-text comments for further explanations where available. QUIS7 was chosen for its simplicity to employ, with minimal training, the areas of usability that could be investigated with participants and its multilingual availability. The usability study aimed to complement other types of feedback received during the platform development phase from the project CRG, as well as project team members. User satisfaction is measured in the QUIS7 questionnaire on a 9-point scale in the following aspects: overall reaction, screen, terminology and tool feedback, learning, multimedia, training material and system capabilities. The 9-point scale ranges from 1, representing a negative adjective, to 9, representing a positive adjective. The QUIS7 questionnaire for patients and informal caregivers is provided in Additional file 9. A link to an online questionnaire was made available to participants via Qualtrics. The QUIS7 questionnaire was available in English, German and Spanish. Translations from English were performed by the corresponding pilot sites where they deemed that the participants in their sites would require it.

The following workshops were held for the PEP usability study: (i) Germany: 1 workshop with 3 patients; (ii) Spain: 1 workshop with 4 patients and 3 carers; and (iii) UK-England: 2 workshops were held online with the first one being a live demonstration of the tool and the second one playing back the recording of the first demonstration. Both meetings were attended by 5 people (Workshop 1: 3 patients and 2 carers; Workshop 2: 4 carers and 1 patient). 18 responses were received in total (Germany 3, Spain 5, UK-England 10): Germany (3 patients), Spain (2 patients, 3 carers), UK-England (3 patients, 6 carers, 1 patient/carer).

As the UK-England site has conducted the usability study using a PPI approach and participants were unable to test the platform themselves, the UK-England site responses have been analyzed separately from the other two sites (Germany and Spain).

## Results

The usability study result data has been analyzed as follows: (i) calculating summary statistics (mean, standard deviation, median) for each questionnaire item for both groups; (ii) presenting of questionnaire results question category on a diverging stacked bar chart for only the group of participants who have tested PEP. The results are presented per category on a diverging stacked bar chart of ranked responses to specially highlight the items that have received the lower scores. These items have been the focus of efforts to improve those aspects of the tools. The comments elaborating on scores given are also assessed to support in the study planning, such as training materials and communication channels. The PPI feedback from the UK-England site provided insight into how the ADLIFE Toolbox can fit within the wider context of healthcare and technology.

For the German and Spanish sites questionnaire results, the average scores for the 7 categories were as follows: Overall reaction 6.77, Screen 7.79, Terminology and Tool Feedback 7.16, Learning 8.38, Multimedia 7.80, Training Material 7.48, System Capabilities 7.64. For the UK-England site, the average scores for the 7 categories were as follows: Overall reaction 6.02, Screen 6.04, Terminology and Tool Feedback 5.89, Learning 5.57, Multimedia 6.34, Training Material 5.41, System Capabilities 5.75. The full set of responses with summary statistics is provided in Additional file 10.

**Fig. 9:**
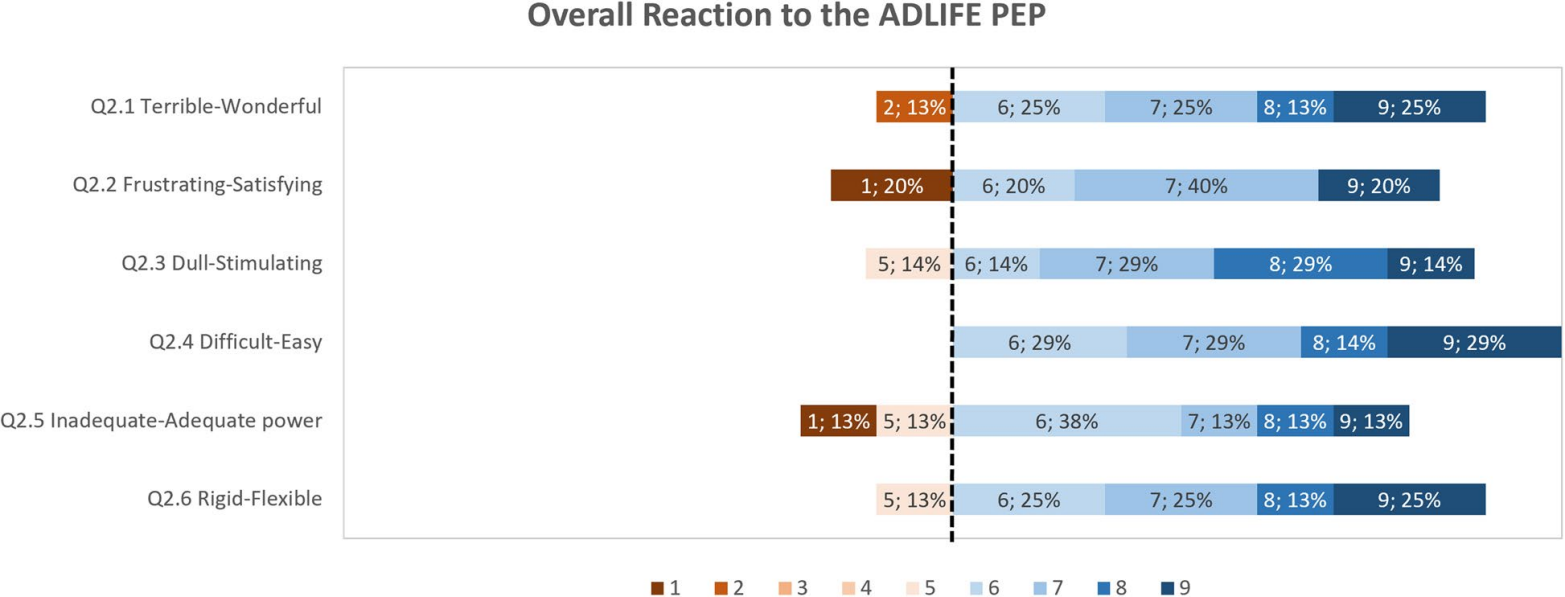
Responses for the PEP QUIS category "Overall reaction"

**Fig. 10:**
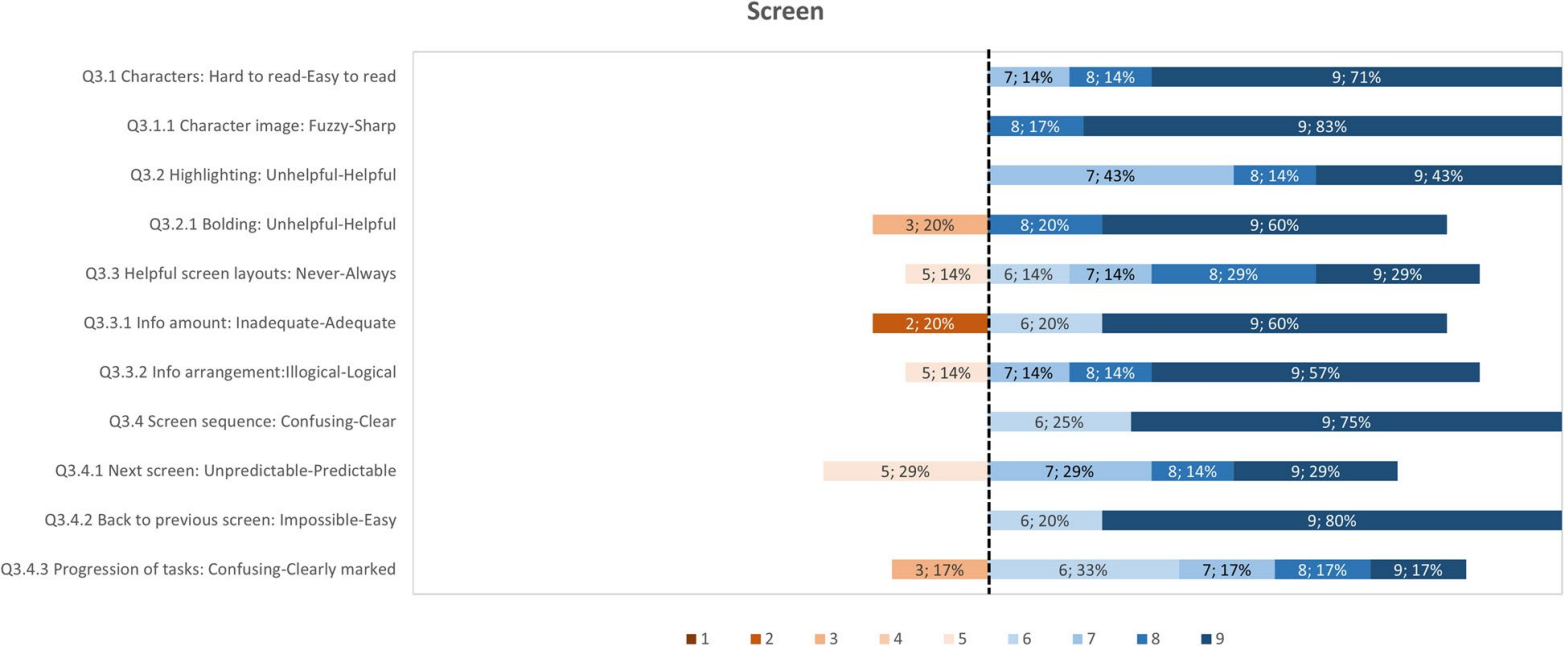
Responses for the PEP QUIS category "Screen"

**Fig. 11:**
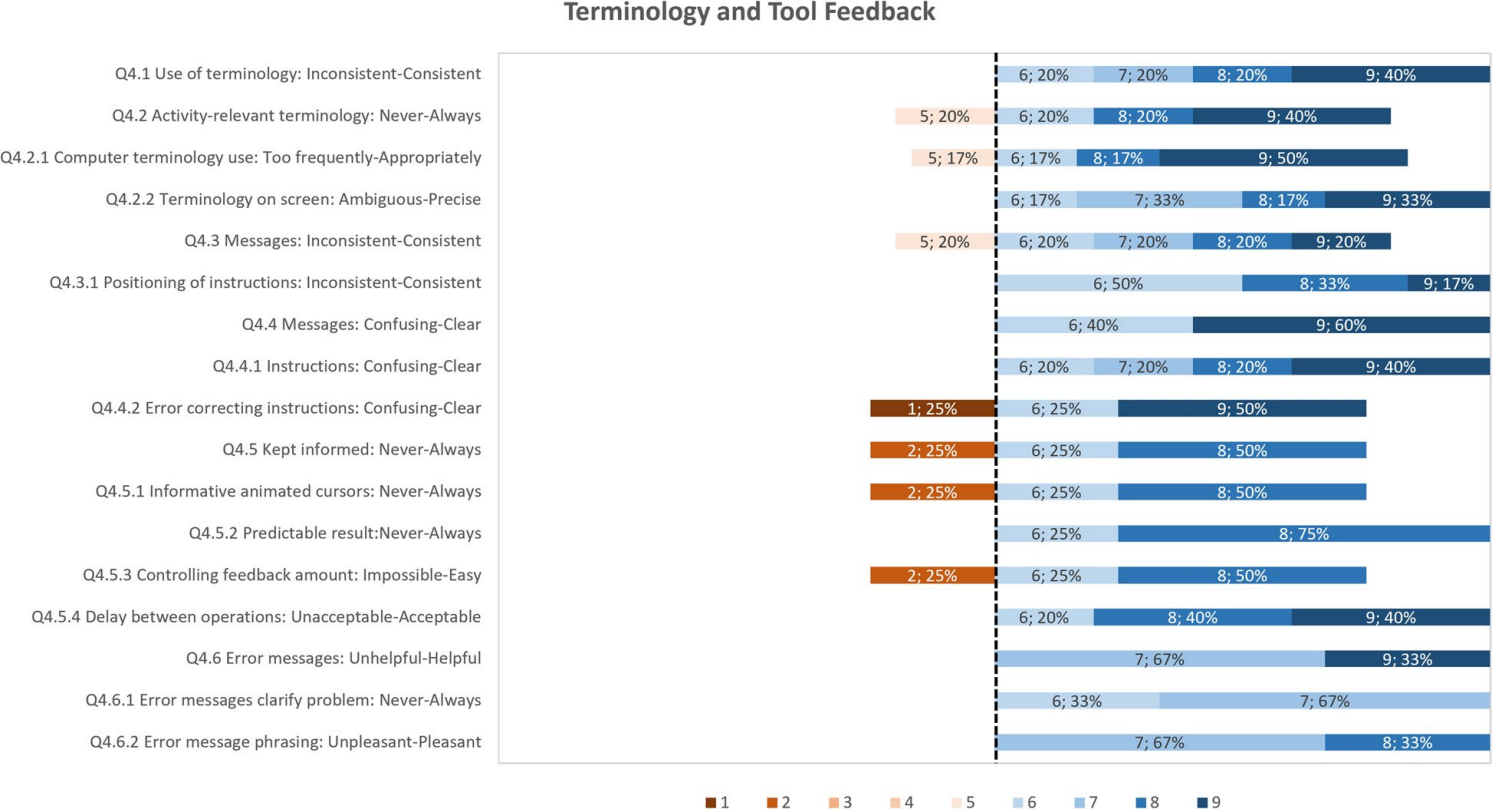
Responses for the PEP QUIS category "Terminology and Tool Feedback"

**Fig. 12:**
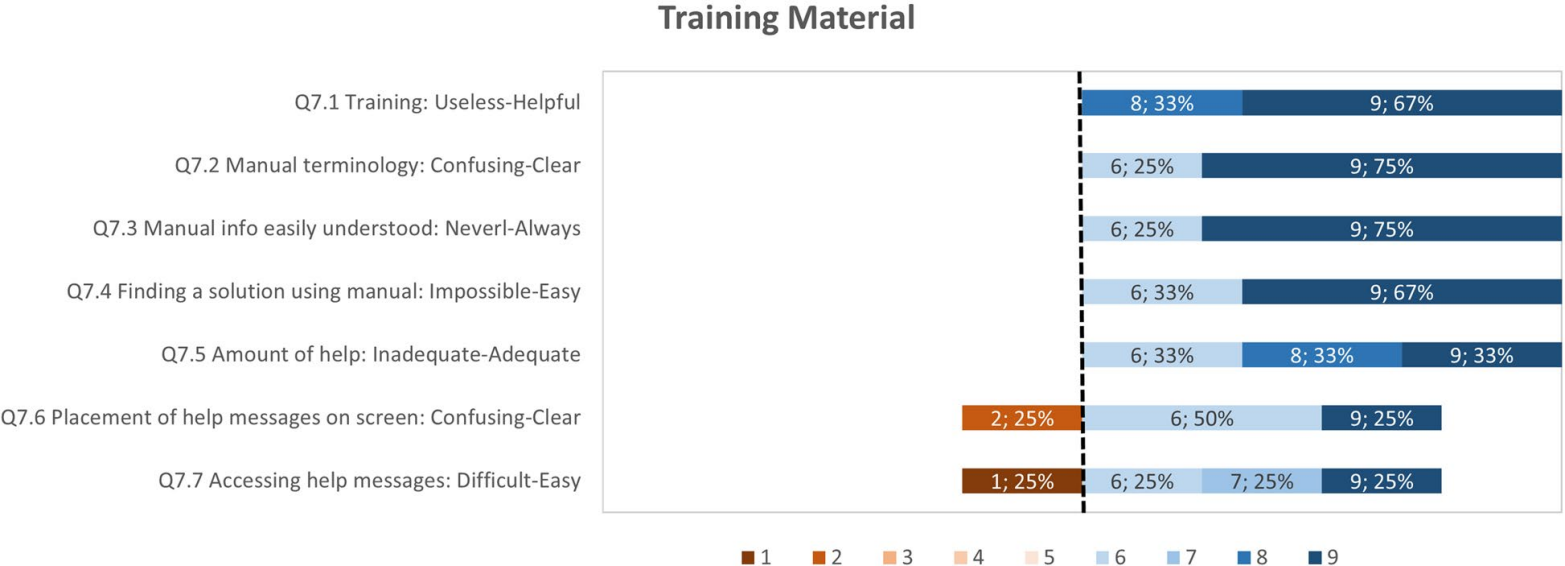
Responses for the PEP QUIS category "Training Material"

In the stacked bar charts for the German and Spanish site results, the score highlighted as the divergent point is 6, as the average score for all categories was above 6. Each questionnaire item is shown as a horizontal bar with the percentage responses for scores from 1 to 9. The data label corresponds to the score, followed by the response rate, e.g. “6, 15%”, meaning that 15% of respondents have selected a score of 6 for that question. Any comments related to specific aspects are included for context. The results for the key categories are presented in Figs. 9 to 12. With all the average scores above 6 in all categories, most respondents have a positive reaction to the ADLIFE PEP platform and find it easy to use. We highlight some of the categories where some of the items have received lower scores for prioritizing updates to the platform where possible. The screen category covered the display and navigation of the tool, with a mean score of 7.79 (Fig. 10). Most respondents have scored the questionnaire items highly. The two items that have received the lowest average scores are those related to the clarity of the progression of tasks and the predictability of the next screen. The mean score for the Terminology and Tool Feedback category is 7.16 (Fig. 11). The questionnaire items that have received the lowest scores relate to how respondents felt that the system kept them informed with feedback and how to correct errors (Q4.5, Q4.5.1, Q4.5.3, Q4.4.2). The Training Material category received a mean score of 7.48, highlighting the importance of training to support end users (Fig. 12). The questionnaire items that have received the lowest scores and less than the diverging point of 6 are those related to the access and placement of help messages on the screen (Q7.6, Q7.7).

The following updates to the ADLIFE PEP platform have been performed as a result of the usability study and complementing feedback from the project:

• First of all, as some of the low-scoring questions were related with ease of navigation of the tool by the patient (See Fig. 10, where some users have commented on screen layouts, and reported that the next screen is unpredictable, progression is unclear), we have implemented a new dashboard user interface. Although patients can view their whole care plan via PEP, following up the routine activities may become difficult with an increasing number of activities, and patients find it difficult to navigate the care plan and clearly see what needs to be done by them and see the progress. We have implemented a simpler dashboard, as home screen of PEP, where patients can easily see the pending actions (such as medications, appointments, questionnaires) they need to carry out during the day as part of their care plan. From this simpler interface, they are also enabled to easily mark the activities as completed with one click. In order to ease the entering information to the PEP system, we have also integrated Android Speech Recognizer to take voice inputs for recording patient data such as the feelings and stress levels of patients.
• To address the negative comments on training material (Fig. 12), the user manuals have been updated to clearly demonstrate how the patient needs to use the system, with clear guidance about navigation among screens. We have also prepared a sample walkthrough scenario, and created videos to depict how the system can be used. These are made available to the users via help screens. On top of this, error messages of the systems have been reviewed and updated to address the comments received in relation to tool feedback (Fig. 11).
• Based on the free-text feedback received from pilot sites as part of usability studies, we have implemented an additional feature to enable the patients to record their medications, which are not listed in their current care plan. These become visible to the healthcare professionals via PCPMP. In addition to this, it has been understood that the medications listed under care plan can be misleading for the patients, as it includes only the medications prescribed within the scope of this specific care plan. To avoid confusion, we have added a new feature to the system to list all prescribed medications of the patient in a separate view, by clearly indicating this view lists all the medications of the patient.
• After usability studies, pilot sites have reported that due to wording of the questionnaires, we should not allow assigning Barthel-Index and IADL questionnaires to patients. We have disabled the assignment of these questionnaires to the patients via PCPMP. These can be completed by the healthcare professionals via PCPMP. In addition to this, CRG group has reviewed the terminology used in PEP screens to address the comments in relation to terminology used in screens (See Fig. 11), and several updates have been carried out to ensure that the terms used are patient friendly.

## Discussion

ADLIFE provides digital health solutions to support personalized, integrated care for chronic disease patients, that enables personalized care plans that are created as a result of cooperation between healthcare professionals and patients facilitated via PROMs and SDM mechanisms. The patient empowerment tools enable the healthcare professionals to collect feedback from the patient via PROMs and questionnaires about their symptoms and general well-being, and to be informed about the preferences of the patient via SDM tools which helps the healthcare professionals to adapt the care plan accordingly. In addition to this, PROMs enabled via PEP and PCPMP will also assist us in evaluation of the clinical impact of ADLIFE digital tools as a result of the ADLIFE clinical pilot study.

The initial usability study indicates a positive overall user satisfaction regarding the ADLIFE PEP platform, which has been well received by participants. By involving a clinical reference group (CRG) composed of GPs, specialists and nurses who are working closely with patients in the development process, we have ensured that the ADLIFE PEP platform addresses the needs and preferences of our end users. We have also tested the usability of our tools with the involvement of patients and informal care givers. Several improvements have been carried out to address the feedback received as summarized in the Results section. This approach aligns with existing knowledge highlighting the importance of user involvement in ensuring a successful adoption of new healthcare technologies in clinical settings [48].

As part of this patient-centered care pathway, the SHARE workflow aims to be a first step towards promoting SDM and involving the patient in treatment decisions. It focuses mainly on offering a structured methodology and support materials to the healthcare professional.

The ‘Ask Three Questions’ decision aid that integrated into the ADLIFE digital toolbox is a good example of a tool that allows in a simple step the SDM approach. It encourages patients to ask three simple questions to lead clinicians to provide higher quality information about treatment options and their benefits and harms. On top of this, the CRG prepared a specific decision aid for ‘shared decision-making on inhalation medicine in patients with COPD’ in order to assess which inhalation medication will best suit the patient, asking the patient to assess what is most important to them and what matters less. This aid has been implemented based on HL7 FHIR and integrated into physician and patient portals.

These decision aids implemented via ADLIFE digital tools supports in a helpful way how professionals and patients can be involved in a clinical decision, considering both the professional and scientific angle, as well as the patient’s values. This approach can improve self-management and adherence, not only medicines management but also factors related to health habits. Personalized educational materials are offered and available for the patient in the PEP, such as diet, exercise, self-monitoring, and participation in self-management education courses.

While the usability study provided valuable insights, it is important to acknowledge certain limitations. The study involved a relatively small sample size, consisting of 20 participants across multiple sites, which restricts the generalizability of the findings. However, the clinical pilot studies address this limitation by involving a larger and more diverse sample size for ADLIFE pilot studies, which will provide a broader range of perspectives. Furthermore, the primary focus of the usability study was on user satisfaction and usability, measured by the QUIS7 questionnaire. While user satisfaction is an important aspect to consider, it does not capture all dimensions of the user experience or the long-term impact of the platform on patient outcomes. To address this, the clinical pilot studies will incorporate additional measures, such as efficiency and effectiveness, to provide a more comprehensive evaluation of the ADLIFE PEP platform in the scope of the ADLIFE Pilot study. ADLIFE pilot study will be conducted in six pilot sites for a duration of 9 months, with the involvement of a total of 1692 patients (846 patients in both control group and intervention group) and will be finalized in the first half of 2024. The details of the study protocol can be found in [19].

Despite these limitations, the usability study conducted for the ADLIFE PEP platform offered valuable insights into user satisfaction, identified areas for improvement, and highlighted the importance of involving end users in the development process. These findings contribute to the growing interest in user involvement in research designs and usability evaluation within healthcare technology, paving the way for continued advancements within the field.

## Conclusions

ADLIFE digital toolbox, composed of two main portals for healthcare professionals and patients, aims to achieve maintenance of a patient-provider partnership as an integrated system of collaborative care, supporting self-management, shared-decision making, collection of patient-reported outcome measures, education, and follow-up.

The usability study demonstrated the importance of involving users in the development of the technical solution. Consequently, the usability study resulted in an adjustment of the technical solution to make the platform more user-friendly for patients. SDM is a newer initiative that has not yet been implemented widely, so it is important to incorporate a reminder in the platform for healthcare professionals to involve patients in all decisions. Having finalized design, implementation, and pre-deployment usability studies, and updated the tool based on further feedback, our patient empowerment mechanisms enabled via PROMs and shared decision-making processes are ready to be piloted in clinal settings. Clinical studies will be conducted based on ADLIFE study protocol [19], at six healthcare settings across Spain, UK, Germany, Denmark, and Israel. After this, a detailed evaluation of user experience, effectiveness, technology acceptance, and socio-economic impact of ADLIFE study will be conducted.

### Supplementary Information 

Additional file 1.

Additional file 2.

Additional file 3.

Additional file 4.

Additional file 5.

Additional file 6.

Additional file 7.

Additional file 8.

Additional file 9.

Additional file 10.

Additional file 11.

Additional file 12.

## Data Availability

All data generated or analyzed during this study are included in this published article and its supplementary information files.

## Abbreviations

AST: Alcohol Screening Tool
CAT: COPD Assessment Test
CDSS: Clinical Decision Support Systems
CHF: Chronic Heart Failure
COPD: Chronic Obstructive Pulmonary Disease
CRG: Clinical Reference Group
JITAIs: Just-in-Time Adaptive Interventions
EHR: Electronic Health Records
EQ-5D-5L: The 5-level version of EuroQol five-dimension scale questionnaire
FAST: Fast Alcohol Screening Tool
FHIR: Fast Healthcare Interoperability Resources
GDS: Global Depression Scale
HADS: Hospital Anxiety and Depression Scale
IADL: Lawton Instrumental Activities of Daily Living Scale
ICHOM: International Consortium for Health Outcome Measures
KCCQ: Kansas City Cardiomyopathy Questionnaire
MADRS: Montgomery and Asberg Depression Rating Scale
mMRC: Modified Medical Research Council Dyspnea Scale
PCQ-P: Person-centered Climate Questionnaire Patient version
PCPMP: Personalized Care Plan Management Platform
PEP: Patient Empowerment Platform
PPI: Patient and Public Involvement
PROM: Patient Reported Outcome Measurement
QUIS7: Questionnaire for User Interface Satisfaction v7
SDM: Shared Decision-making
WEMWBS: Warwick Medical School Wellbeing questionnaire
ZBI-22: Zarit Burden Interview: 12-item version
