# Supplementary material for: Design, implementation and usability analysis of patient empowerment in ADLIFE project via patient reported outcome measures and shared decision making
Source: BMC Med Inform Decis Mak. 2024 Jun 28;24:185. doi: 10.1186/s12911-024-02588-y (PMC11212241; doi:10.1186/s12911-024-02588-y)
Supplement: Supplementary file 1 — Additional file 1. [file 12911_2024_2588_MOESM1_ESM.rtf]

 Additional File 1a.     File format: . rtfb.     Title: Kansas City Cardiomyopathy Questionnaire (KCCQ) FHIR Resourcec.     Description of Data: HL7 FHIR Representation of Kansas City Cardiomyopathy Questionnaire (KCCQ) as a Questionnaire Resource instance{    "resourceType": "Questionnaire",    "id": "q-kccq",    "language": "gb",    "meta":    {        "tag":        [            {                "system": "http://kroniq.srdc.com.tr/fhir/CodeSystem/questionnaire-category",                "code": "patient"            }        ]    },    "extension":    [        {            "url": "http://adlifeproject.com/fhir/StructureDefinition/health-outcome-area",            "valueCodeableConcept":            {                "coding":                [                    {                        "system": "http://adlifeproject.com/fhir/CodeSystem/health-outcome-area",                        "code": "symptoms-functioning-qol"                    }                ]            }        },        {            "url": "http://adlifeproject.com/fhir/StructureDefinition/health-outcome-dimension",            "valueCodeableConcept":            {                "coding":                [                    {                        "system": "http://adlifeproject.com/fhir/CodeSystem/health-outcome-dimension",                        "code": "activities-of-daily-living"                    }                ]            }        }    ],    "title": "Kansas City Cardiomyopathy Questionnaire",    "status": "active",    "subjectType":    [        "Patient"    ],    "date": "2022-11-30T11:00:00+03:00",    "code":    [        {            "system": "http://loinc.org",            "code": "86924-8",            "display": "Kansas City Cardiomyopathy Questionnaire"        }    ],    "item":    [        {            "linkId": "questionnaire-kccq-section-1",            "type": "group",            "text": "Heart failure affects different people in different ways. Some may mainly feel shortness of breath while others mainly fatigue. Please indicate how limited you have been by heart failure (for example, shortness of breath or fatigue) in your ability to do the following activities over the past 2 weeks.",            "item":            [                {                    "linkId": "questionnaire-kccq-1-1",                    "text": "Dressing yourself",                    "type": "choice",                    "required": true,                    "answerOption":                    [                        {                            "valueCoding":                            {                                "code": "0",                                "display": "Extremely Limited"                            }                        },                        {                            "valueCoding":                            {                                "code": "1",                                "display": "Quite a bit Limited"                            }                        },                        {                            "valueCoding":                            {                                "code": "2",                                "display": "Moderately Limited"                            }                        },                        {                            "valueCoding":                            {                                "code": "3",                                "display": "Slightly Limited"                            }                        },                        {                            "valueCoding":                            {                                "code": "4",                                "display": "Not at all Limited"                            }                        },                        {                            "valueCoding":                            {                                "code": "5",                                "display": "Limited for other reasons or did not do the activity"                            }                        }                    ]                },                {                    "linkId": "questionnaire-kccq-1-2",                    "text": "Showering or having a bath",                    "type": "choice",                    "required": true,                    "answerOption":                    [                        {                            "valueCoding":                            {                                "code": "0",                                "display": "Extremely Limited"                            }                        },                        {                            "valueCoding":                            {                                "code": "1",                                "display": "Quite a bit Limited"                            }                        },                        {                            "valueCoding":                            {                                "code": "2",                                "display": "Moderately Limited"                            }                        },                        {                            "valueCoding":                            {                                "code": "3",                                "display": "Slightly Limited"                            }                        },                        {                            "valueCoding":                            {                                "code": "4",                                "display": "Not at all Limited"                            }                        },                        {                            "valueCoding":                            {                                "code": "5",                                "display": "Limited for other reasons or did not do the activity"                            }                        }                    ]                },                {                    "linkId": "questionnaire-kccq-1-3",                    "text": "Walking 100 yards on level ground",                    "type": "choice",                    "required": true,                    "answerOption":                    [                        {                            "valueCoding":                            {                                "code": "0",                                "display": "Extremely Limited"                            }                        },                        {                            "valueCoding":                            {                                "code": "1",                                "display": "Quite a bit Limited"                            }                        },                        {                            "valueCoding":                            {                                "code": "2",                                "display": "Moderately Limited"                            }                        },                        {                            "valueCoding":                            {                                "code": "3",                                "display": "Slightly Limited"                            }                        },                        {                            "valueCoding":                            {                                "code": "4",                                "display": "Not at all Limited"                            }                        },                        {                            "valueCoding":                            {                                "code": "5",                                "display": "Limited for other reasons or did not do the activity"                            }                        }                    ]                },                {                    "linkId": "questionnaire-kccq-1-4",                    "text": "Doing gardening, housework or carrying groceries",                    "type": "choice",                    "required": true,                    "answerOption":                    [                        {                            "valueCoding":                            {                                "code": "0",                                "display": "Extremely Limited"                            }                        },                        {                            "valueCoding":                            {                                "code": "1",                                "display": "Quite a bit Limited"                            }                        },                        {                            "valueCoding":                            {                                "code": "2",                                "display": "Moderately Limited"                            }                        },                        {                            "valueCoding":                            {                                "code": "3",                                "display": "Slightly Limited"                            }                        },                        {                            "valueCoding":                            {                                "code": "4",                                "display": "Not at all Limited"                            }                        },                        {                            "valueCoding":                            {                                "code": "5",                                "display": "Limited for other reasons or did not do the activity"                            }                        }                    ]                },                {                    "linkId": "questionnaire-kccq-1-5",                    "text": "Climbing a flight of stairs without stopping",                    "type": "choice",                    "required": true,                    "answerOption":                    [                        {                            "valueCoding":                            {                                "code": "0",                                "display": "Extremely Limited"                            }                        },                        {                            "valueCoding":                            {                                "code": "1",                                "display": "Quite a bit Limited"                            }                        },                        {                            "valueCoding":                            {                                "code": "2",                                "display": "Moderately Limited"                            }                        },                        {                            "valueCoding":                            {                                "code": "3",                                "display": "Slightly Limited"                            }                        },                        {                            "valueCoding":                            {                                "code": "4",                                "display": "Not at all Limited"                            }                        },                        {                            "valueCoding":                            {                                "code": "5",                                "display": "Limited for other reasons or did not do the activity"                            }                        }                    ]                },                {                    "linkId": "questionnaire-kccq-1-6",                    "text": "Jogging or hurrying (as if to catch a bus)",                    "type": "choice",                    "required": true,                    "answerOption":                    [                        {                            "valueCoding":                            {                                "code": "0",                                "display": "Extremely Limited"                            }                        },                        {                            "valueCoding":                            {                                "code": "1",                                "display": "Quite a bit Limited"                            }                        },                        {                            "valueCoding":                            {                                "code": "2",                                "display": "Moderately Limited"                            }                        },                        {                            "valueCoding":                            {                                "code": "3",                                "display": "Slightly Limited"                            }                        },                        {                            "valueCoding":                            {                                "code": "4",                                "display": "Not at all Limited"                            }                        },                        {                            "valueCoding":                            {                                "code": "5",                                "display": "Limited for other reasons or did not do the activity"                            }                        }                    ]                }            ]        },        {            "linkId": "questionnaire-kccq-section-2",            "type": "group",            "item":            [                {                    "linkId": "questionnaire-kccq-2-1",                    "text": "Compared with 2 weeks ago, have your symptoms of heart failure (for example, shortness of breath, fatigue, or ankle swelling) changed? My symptoms of heart failure are now…",                    "type": "choice",                    "required": true,                    "answerOption":                    [                        {                            "valueCoding":                            {                                "code": "0",                                "display": "Much worse"                            }                        },                        {                            "valueCoding":                            {                                "code": "1",                                "display": "Slightly worse"                            }                        },                        {                            "valueCoding":                            {                                "code": "2",                                "display": "Not changed"                            }                        },                        {                            "valueCoding":                            {                                "code": "3",                                "display": "Slightly better"                            }                        },                        {                            "valueCoding":                            {                                "code": "4",                                "display": "Much better"                            }                        },                        {                            "valueCoding":                            {                                "code": "5",                                "display": "I've had no symptoms over the last 2 weeks"                            }                        }                    ]                },                {                    "linkId": "questionnaire-kccq-2-2",                    "text": "Over the past 2 weeks, how many times have you had swelling in your feet, ankles or legs when you woke up in the morning?",                    "type": "choice",                    "required": true,                    "answerOption":                    [                        {                            "valueCoding":                            {                                "code": "0",                                "display": "Every morning"                            }                        },                        {                            "valueCoding":                            {                                "code": "1",                                "display": "3 or more times a week, but not every day"                            }                        },                        {                            "valueCoding":                            {                                "code": "2",                                "display": "1-2 times a week"                            }                        },                        {                            "valueCoding":                            {                                "code": "3",                                "display": "Less than once a week"                            }                        },                        {                            "valueCoding":                            {                                "code": "4",                                "display": "Never over the past 2 weeks"                            }                        }                    ]                },                {                    "linkId": "questionnaire-kccq-2-3",                    "text": "Over the past 2 weeks, how much has swelling in your feet, ankles or legs bothered you?",                    "type": "choice",                    "required": true,                    "answerOption":                    [                        {                            "valueCoding":                            {                                "code": "0",                                "display": "Extremely bothersome"                            }                        },                        {                            "valueCoding":                            {                                "code": "1",                                "display": "Quite a bit bothersome"                            }                        },                        {                            "valueCoding":                            {                                "code": "2",                                "display": "Moderately bothersome"                            }                        },                        {                            "valueCoding":                            {                                "code": "3",                                "display": "Slightly bothersome"                            }                        },                        {                            "valueCoding":                            {                                "code": "4",                                "display": "Not at all bothersome"                            }                        },                        {                            "valueCoding":                            {                                "code": "5",                                "display": "I've had no swelling"                            }                        }                    ]                },                {                    "linkId": "questionnaire-kccq-2-4",                    "text": "Over the past 2 weeks, on average, how many times has fatigue limited your ability to do what you wanted?",                    "type": "choice",                    "required": true,                    "answerOption":                    [                        {                            "valueCoding":                            {                                "code": "0",                                "display": "All of the time"                            }                        },                        {                            "valueCoding":                            {                                "code": "1",                                "display": "Several times per day"                            }                        },                        {                            "valueCoding":                            {                                "code": "2",                                "display": "At least once a day"                            }                        },                        {                            "valueCoding":                            {                                "code": "3",                                "display": "3 or more times per week but not every day"                            }                        },                        {                            "valueCoding":                            {                                "code": "4",                                "display": "1-2 times per week"                            }                        },                        {                            "valueCoding":                            {                                "code": "5",                                "display": "Less than once a week"                            }                        },                        {                            "valueCoding":                            {                                "code": "6",                                "display": "Never over the past 2 weeks"                            }                        }                    ]                },                {                    "linkId": "questionnaire-kccq-2-5",                    "text": "Over the past 2 weeks, how much has your fatigue bothered you?",                    "type": "choice",                    "required": true,                    "answerOption":                    [                        {                            "valueCoding":                            {                                "code": "0",                                "display": "Extremely bothersome"                            }                        },                        {                            "valueCoding":                            {                                "code": "1",                                "display": "Quite a bit bothersome"                            }                        },                        {                            "valueCoding":                            {                                "code": "2",                                "display": "Moderately bothersome"                            }                        },                        {                            "valueCoding":                            {                                "code": "3",                                "display": "Slightly bothersome"                            }                        },                        {                            "valueCoding":                            {                                "code": "4",                                "display": "Not at all bothersome"                            }                        },                        {                            "valueCoding":                            {                                "code": "5",                                "display": "I've had no fatigue"                            }                        }                    ]                },                {                    "linkId": "questionnaire-kccq-2-6",                    "text": "Over the past 2 weeks, on average, how many times has shortness of breath limited your ability to do what you wanted?",                    "type": "choice",                    "required": true,                    "answerOption":                    [                        {                            "valueCoding":                            {                                "code": "0",                                "display": "All of the time"                            }                        },                        {                            "valueCoding":                            {                                "code": "1",                                "display": "Several times a day"                            }                        },                        {                            "valueCoding":                            {                                "code": "2",                                "display": "At least once a day"                            }                        },                        {                            "valueCoding":                            {                                "code": "3",                                "display": "3 or more times a week but not every day"                            }                        },                        {                            "valueCoding":                            {                                "code": "4",                                "display": "1-2 times a week"                            }                        },                        {                            "valueCoding":                            {                                "code": "5",                                "display": "Less than once a week"                            }                        },                        {                            "valueCoding":                            {                                "code": "6",                                "display": "Never over the past 2 weeks"                            }                        }                    ]                },                {                    "linkId": "questionnaire-kccq-2-7",                    "text": "Over the past 2 weeks, how much has your shortness of breath bothered you?",                    "type": "choice",                    "required": true,                    "answerOption":                    [                        {                            "valueCoding":                            {                                "code": "0",                                "display": "Extremely bothersome"                            }                        },                        {                            "valueCoding":                            {                                "code": "1",                                "display": "Quite a bit bothersome"                            }                        },                        {                            "valueCoding":                            {                                "code": "2",                                "display": "Moderately bothersome"                            }                        },                        {                            "valueCoding":                            {                                "code": "3",                                "display": "Slightly bothersome"                            }                        },                        {                            "valueCoding":                            {                                "code": "4",                                "display": "Not at all bothersome"                            }                        },                        {                            "valueCoding":                            {                                "code": "5",                                "display": "I’ve had no shortness of breath"                            }                        }                    ]                },                {                    "linkId": "questionnaire-kccq-2-8",                    "text": "Over the past 2 weeks, on average, how many times have you been forced to sleep sitting up in a chair or with at least 3 pillows to prop you up because of shortness of breath?",                    "type": "choice",                    "required": true,                    "answerOption":                    [                        {                            "valueCoding":                            {                                "code": "0",                                "display": "Every night"                            }                        },                        {                            "valueCoding":                            {                                "code": "1",                                "display": "3 or more times a week, but not every night"                            }                        },                        {                            "valueCoding":                            {                                "code": "2",                                "display": "1-2 times a week"                            }                        },                        {                            "valueCoding":                            {                                "code": "3",                                "display": "Less than once a week"                            }                        },                        {                            "valueCoding":                            {                                "code": "4",                                "display": "Never over the past 2 weeks"                            }                        }                    ]                },                {                    "linkId": "questionnaire-kccq-2-9",                    "text": "Heart failure symptoms can worsen for a number of reasons. How sure are you that you know what to do, or whom to call, if your heart failure gets worse?",                    "type": "choice",                    "required": true,                    "answerOption":                    [                        {                            "valueCoding":                            {                                "code": "0",                                "display": "Not at all sure"                            }                        },                        {                            "valueCoding":                            {                                "code": "1",                                "display": "Not very sure"                            }                        },                        {                            "valueCoding":                            {                                "code": "2",                                "display": "Somewhat sure"                            }                        },                        {                            "valueCoding":                            {                                "code": "3",                                "display": "Mostly sure"                            }                        },                        {                            "valueCoding":                            {                                "code": "4",                                "display": "Completely sure"                            }                        }                    ]                },                {                    "linkId": "questionnaire-kccq-2-10",                    "text": "How well do you understand what things you are able to do to keep your heart failure symptoms from getting worse (for example, regularly weighing yourself, eating a low salt diet etc.)?",                    "type": "choice",                    "required": true,                    "answerOption":                    [                        {                            "valueCoding":                            {                                "code": "0",                                "display": "Do not understand at all"                            }                        },                        {                            "valueCoding":                            {                                "code": "1",                                "display": "Do not understand very well"                            }                        },                        {                            "valueCoding":                            {                                "code": "2",                                "display": "Somewhat understand"                            }                        },                        {                            "valueCoding":                            {                                "code": "3",                                "display": "Mostly understand"                            }                        },                        {                            "valueCoding":                            {                                "code": "4",                                "display": "Completely understand"                            }                        }                    ]                },                {                    "linkId": "questionnaire-kccq-2-11",                    "text": "Over the past 2 weeks, how much has your heart failure limited your enjoyment of life?",                    "type": "choice",                    "required": true,                    "answerOption":                    [                        {                            "valueCoding":                            {                                "code": "0",                                "display": "It has extremely limited my enjoyment of life"                            }                        },                        {                            "valueCoding":                            {                                "code": "1",                                "display": "It has limited my enjoyment of life quite a bit"                            }                        },                        {                            "valueCoding":                            {                                "code": "2",                                "display": "It has moderately limited my enjoyment of life"                            }                        },                        {                            "valueCoding":                            {                                "code": "3",                                "display": "It has slightly limited my enjoyment of life"                            }                        },                        {                            "valueCoding":                            {                                "code": "4",                                "display": "It has not limited my enjoyment of life at all"                            }                        }                    ]                },                {                    "linkId": "questionnaire-kccq-2-12",                    "text": "If you had to spend the rest of your life with your heart failure the way it is right now, how would you feel about this?",                    "type": "choice",                    "required": true,                    "answerOption":                    [                        {                            "valueCoding":                            {                                "code": "0",                                "display": "Completely dissatisfied"                            }                        },                        {                            "valueCoding":                            {                                "code": "1",                                "display": "Mostly dissatisfied"                            }                        },                        {                            "valueCoding":                            {                                "code": "2",                                "display": "Somewhat satisfied"                            }                        },                        {                            "valueCoding":                            {                                "code": "3",                                "display": "Mostly satisfied"                            }                        },                        {                            "valueCoding":                            {                                "code": "4",                                "display": "Completely satisfied"                            }                        }                    ]                },                {                    "linkId": "questionnaire-kccq-2-13",                    "text": "Over the past 2 weeks, how often have you felt discouraged or down in the dumps because of your heart failure?",                    "type": "choice",                    "required": true,                    "answerOption":                    [                        {                            "valueCoding":                            {                                "code": "0",                                "display": "I have felt that way all of the time"                            }                        },                        {                            "valueCoding":                            {                                "code": "1",                                "display": "I have felt that way most of the time"                            }                        },                        {                            "valueCoding":                            {                                "code": "2",                                "display": "I have occasionally felt that way"                            }                        },                        {                            "valueCoding":                            {                                "code": "3",                                "display": "I have rarely felt that way"                            }                        },                        {                            "valueCoding":                            {                                "code": "4",                                "display": "I have never felt that way"                            }                        }                    ]                }            ]        },        {            "linkId": "questionnaire-kccq-section-3",            "type": "group",            "text": "How much does your heart failure affect your lifestyle? Please indicate how your heart failure may have limited your participation in the following activities over the past 2 weeks.",            "item":            [                {                    "linkId": "questionnaire-kccq-3-1",                    "text": "Hobbies,recreational activities",                    "type": "choice",                    "required": true,                    "answerOption":                    [                        {                            "valueCoding":                            {                                "code": "0",                                "display": "Extremely limited"                            }                        },                        {                            "valueCoding":                            {                                "code": "1",                                "display": "Quite a bit limited"                            }                        },                        {                            "valueCoding":                            {                                "code": "2",                                "display": "Moderately limited"                            }                        },                        {                            "valueCoding":                            {                                "code": "3",                                "display": "Slightly limited"                            }                        },                        {                            "valueCoding":                            {                                "code": "4",                                "display": "Not at all limited"                            }                        },                        {                            "valueCoding":                            {                                "code": "5",                                "display": "Limited for other reasons or did not do the activity"                            }                        }                    ]                },                {                    "linkId": "questionnaire-kccq-3-2",                    "text": "Working or doing household chores",                    "type": "choice",                    "required": true,                    "answerOption":                    [                        {                            "valueCoding":                            {                                "code": "0",                                "display": "Extremely limited"                            }                        },                        {                            "valueCoding":                            {                                "code": "1",                                "display": "Quite a bit limited"                            }                        },                        {                            "valueCoding":                            {                                "code": "2",                                "display": "Moderately limited"                            }                        },                        {                            "valueCoding":                            {                                "code": "3",                                "display": "Slightly limited"                            }                        },                        {                            "valueCoding":                            {                                "code": "4",                                "display": "Not at all limited"                            }                        },                        {                            "valueCoding":                            {                                "code": "5",                                "display": "Limited for other reasons or did not do the activity"                            }                        }                    ]                },                {                    "linkId": "questionnaire-kccq-3-3",                    "text": "Visiting family or friends",                    "type": "choice",                    "required": true,                    "answerOption":                    [                        {                            "valueCoding":                            {                                "code": "0",                                "display": "Extremely limited"                            }                        },                        {                            "valueCoding":                            {                                "code": "1",                                "display": "Quite a bit limited"                            }                        },                        {                            "valueCoding":                            {                                "code": "2",                                "display": "Moderately limited"                            }                        },                        {                            "valueCoding":                            {                                "code": "3",                                "display": "Slightly limited"                            }                        },                        {                            "valueCoding":                            {                                "code": "4",                                "display": "Not at all limited"                            }                        },                        {                            "valueCoding":                            {                                "code": "5",                                "display": "Limited for other reasons or did not do the activity"                            }                        }                    ]                },                {                    "linkId": "questionnaire-kccq-3-4",                    "text": "Intimate or sexual relationships",                    "type": "choice",                    "required": true,                    "answerOption":                    [                        {                            "valueCoding":                            {                                "code": "0",                                "display": "Extremely limited"                            }                        },                        {                            "valueCoding":                            {                                "code": "1",                                "display": "Quite a bit limited"                            }                        },                        {                            "valueCoding":                            {                                "code": "2",                                "display": "Moderately limited"                            }                        },                        {                            "valueCoding":                            {                                "code": "3",                                "display": "Slightly limited"                            }                        },                        {                            "valueCoding":                            {                                "code": "4",                                "display": "Not at all limited"                            }                        },                        {                            "valueCoding":                            {                                "code": "5",                                "display": "Limited for other reasons or did not do the activity"                            }                        }                    ]                }            ]        }    ]}
