# Supplementary material for: Design, implementation and usability analysis of patient empowerment in ADLIFE project via patient reported outcome measures and shared decision making
Source: BMC Med Inform Decis Mak. 2024 Jun 28;24:185. doi: 10.1186/s12911-024-02588-y (PMC11212241; doi:10.1186/s12911-024-02588-y)
Supplement: Supplementary file 2 — Additional file 2. [file 12911_2024_2588_MOESM2_ESM.rtf]

Additional File 2a.     File format: . rtfb.     Title: An example CarePlan FHIR Resourcec.     Description of Data: HL7 FHIR Representation of an example care plan that assigns Kansas City Cardiomyopathy Questionnaire (KCCQ) as a patient activity to be performed by the patient. {    "resourceType": "CarePlan",    "id": "b29ed59c-54e4-4d58-9810-36b5ac7c8c41",    "meta":    {        "versionId": "50",        "lastUpdated": "2023-06-09T17:54:19.390+03:00"    },    "subject":    {        "reference": "Patient/6f4cd139-c920-445c-a0e3-e9e60c5ae235",        "display": "Jane Doe"    },    "status": "active",    "intent": "plan",    "title": "Care Plan of Jane Doe",    "author":    {        "reference": "Practitioner/d1bd226f-c4ed-4f15-b366-0d2005d83c47",        "display": "Can Yımaz"    },    "careTeam":    [        {            "reference": "CareTeam/6f4cd139-c920-445c-a0e3-e9e60c5ae235-careTeam",            "display": "Care Team of Jane Doe"        }    ],    "period":    {        "start": "2023-06-09T14:53:47.798Z"    },    "goal":    [        {            "reference": "Goal/5bbaee61-0166-db1e-260f-3b6f62fe1956",            "display": "Cholesterol in LDL [Mass/volume] in Serum or Plasma"        },        {            "reference": "Goal/f44e85d1-e950-f780-cbdc-8ec3b50da9f0",            "display": "Systolic blood pressure"        },        {            "reference": "Goal/ff00f831-8cc9-c922-3d37-d1fec8a4f0fb",            "display": "Diastolic blood pressure"        }    ],    "activity":    [        {            "reference":            {                "reference": "ServiceRequest/8bdcab28-3182-d4ba-49d1-e8227ea2b20c",                "display": "eGFR"            }        },        {            "reference":            {                "reference": "ServiceRequest/d37ada66-fbe7-1d3b-ca15-71bc4c61fcc3",                "display": "Sodium"            }        },        {            "reference":            {                "reference": "ServiceRequest/63051743-55b7-26d3-3579-83b5282482a9",                "display": "Creatinine"            }        },        {            "reference":            {                "reference": "MedicationRequest/f9d2057d-ca08-ba33-bd04-23aad24a66b0",                "display": "Statin"            }        },        {            "reference":            {                "reference": "ServiceRequest/f304953d-314f-4b78-966b-fb81943a314f",                "display": "Kansas City Cardiomyopathy Questionnaire"            }        },        {            "reference":            {                "reference": "ServiceRequest/e887f872-e97a-4c8e-812c-8c53c7b3830e",                "display": "Shared decision-Making on inhalation medicine in patients of COPD"            }        }    ]}{    "resourceType": "ServiceRequest",    "id": "e887f872-e97a-4c8e-812c-8c53c7b3830e",    "meta": {        "versionId": "1",        "lastUpdated": "2023-06-09T17:54:17.391+03:00"    },    "status": "active",    "category": [        {            "coding": [                {                    "system": "http://kroniq.srdc.com.tr/fhir/CodeSystem/care-plan-activity-category",                    "code": "questionnaire",                    "display": "Questionnaire"                }            ]        }    ],    "intent": "order",    "extension": [        {            "url": "http://hl7.org/fhir/StructureDefinition/servicerequest-questionnaireRequest",            "valueReference": {                "reference": "Questionnaire/q-sdmim",                "display": "Shared decision-Making on inhalation medicine in patients of COPD",                "identifier": {                    "system": "http://kroniq.srdc.com.tr/fhir/CodeSystem/questionnaire-code",                    "value": "sdmim"                }            }        },        {            "url": "http://kroniq.srdc.com.tr/fhir/StructureDefinition/title",            "valueString": "Shared decision-Making on inhalation medicine in patients of COPD"        }    ],    "subject": {        "reference": "Patient/6f4cd139-c920-445c-a0e3-e9e60c5ae235",        "display": "Jane Doe"    },    "occurrenceTiming": {        "repeat": {            "boundsPeriod": {                "start": "2023-06-09T14:54:16.296Z",                "end": "2023-07-09T14:54:16.296Z"            }        }    },    "requester": {        "reference": "Practitioner/3c670b96-8657-4fe7-bc48-b4352a2b5284",        "display": "Anna Svensson"    },    "performer": [        {            "reference": "Patient/6f4cd139-c920-445c-a0e3-e9e60c5ae235",            "display": "Jane Doe"        }    ],    "note": [        {            "authorReference": {                "reference": "Practitioner/3c670b96-8657-4fe7-bc48-b4352a2b5284",                "display": "Anna Svensson"            },            "time": "2023-06-09T14:54:17.384Z",            "text": "created the activity.",            "extension": [                {                    "url": "http://kroniq.srdc.com.tr/fhir/StructureDefinition/action-type",                    "valueCode": "create"                }            ]        }    ]}{    "resourceType": "ServiceRequest",    "id": "f304953d-314f-4b78-966b-fb81943a314f",    "meta": {        "versionId": "3",        "lastUpdated": "2023-06-09T18:00:46.461+03:00"    },    "status": "active",    "category": [        {            "coding": [                {                    "system": "http://kroniq.srdc.com.tr/fhir/CodeSystem/care-plan-activity-category",                    "code": "questionnaire",                    "display": "Questionnaire"                }            ]        }    ],    "intent": "order",    "subject": {        "reference": "Patient/6f4cd139-c920-445c-a0e3-e9e60c5ae235",        "display": "Jane Doe"    },    "occurrenceTiming": {        "repeat": {            "boundsPeriod": {                "start": "2023-06-09T14:54:08.935Z",                "end": "2024-06-09T14:54:08.935Z"            },            "frequency": 1,            "period": 1,            "periodUnit": "mo"        }    },    "requester": {        "reference": "Practitioner/3c670b96-8657-4fe7-bc48-b4352a2b5284",        "display": "Anna Svensson"    },    "performer": [        {            "reference": "Patient/6f4cd139-c920-445c-a0e3-e9e60c5ae235",            "display": "Jane Doe"        }    ],    "note": [        {            "authorReference": {                "reference": "Practitioner/3c670b96-8657-4fe7-bc48-b4352a2b5284",                "display": "Anna Svensson"            },            "time": "2023-06-09T15:00:46.449Z",            "text": "updated the activity.",            "extension": [                {                    "url": "http://kroniq.srdc.com.tr/fhir/StructureDefinition/action-type",                    "valueCode": "update"                }            ]        },        {            "authorReference": {                "reference": "Practitioner/3c670b96-8657-4fe7-bc48-b4352a2b5284",                "display": "Anna Svensson"            },            "time": "2023-06-09T15:00:14.522Z",            "text": "updated the activity.",            "extension": [                {                    "url": "http://kroniq.srdc.com.tr/fhir/StructureDefinition/action-type",                    "valueCode": "update"                }            ]        },        {            "authorReference": {                "reference": "Practitioner/3c670b96-8657-4fe7-bc48-b4352a2b5284",                "display": "Anna Svensson"            },            "time": "2023-06-09T14:54:10.538Z",            "text": "created the activity.",            "extension": [                {                    "url": "http://kroniq.srdc.com.tr/fhir/StructureDefinition/action-type",                    "valueCode": "create"                }            ]        }    ],    "extension": [        {            "url": "http://hl7.org/fhir/StructureDefinition/servicerequest-questionnaireRequest",            "valueReference": {                "reference": "Questionnaire/q-kccq",                "display": "Kansas City Cardiomyopathy Questionnaire",                "identifier": {                    "system": "http://loinc.org",                    "value": "86924-8"                }            }        },        {            "url": "http://kroniq.srdc.com.tr/fhir/StructureDefinition/title",            "valueString": "Kansas City Cardiomyopathy Questionnaire"        }    ]}
