# Supplementary material for: Design, implementation and usability analysis of patient empowerment in ADLIFE project via patient reported outcome measures and shared decision making
Source: BMC Med Inform Decis Mak. 2024 Jun 28;24:185. doi: 10.1186/s12911-024-02588-y (PMC11212241; doi:10.1186/s12911-024-02588-y)
Supplement: Supplementary file 3 — Additional file 3. [file 12911_2024_2588_MOESM3_ESM.rtf]

Additional File 3a.     File format: . rtfb.     Title: An example QuestionnaireResponse FHIR Resource for Kansas City Cardiomyopathy Questionnaire (KCCQ)c.     Description of Data: HL7 FHIR Representation of an example questionnaire response for Kansas City Cardiomyopathy Questionnaire (KCCQ) {    "resourceType": "QuestionnaireResponse",    "id": "d0915fc8-823b-4f66-b6b2-6def9271acba",    "meta":    {        "source": "http://kroniq.srdc.com.tr/patient",        "tag":        [            {                "system": "http://kroniq.srdc.com.tr/fhir/CodeSystem/questionnaire-category",                "code": "patient"            }        ],        "versionId": "1",        "lastUpdated": "2023-06-09T19:33:14.406+03:00"    },    "identifier":    {        "system": "http://kroniq.srdc.com.tr/fhir/CodeSystem/questionnaire-code",        "value": "86924-8"    },    "status": "completed",    "subject":    {        "reference": "Patient/6f4cd139-c920-445c-a0e3-e9e60c5ae235"    },    "questionnaire": "Questionnaire/q-kccq",    "language": "gb",    "author":    {        "reference": "Patient/6f4cd139-c920-445c-a0e3-e9e60c5ae235",        "display": "Jane Doe"    },    "authored": "2023-06-09T16:33:13.654Z",    "item":    [        {            "linkId": "questionnaire-kccq-section-1",            "text": "Heart failure affects different people in different ways. Some may mainly feel shortness of breath while others mainly fatigue. Please indicate how limited you have been by heart failure (for example, shortness of breath or fatigue) in your ability to do the following activities over the past 2 weeks.",            "item":            [                {                    "linkId": "questionnaire-kccq-1-1",                    "text": "Dressing yourself",                    "answer":                    [                        {                            "valueCoding":                            {                                "code": "1",                                "display": "Quite a bit Limited"                            }                        }                    ]                },                {                    "linkId": "questionnaire-kccq-1-2",                    "text": "Showering or having a bath",                    "answer":                    [                        {                            "valueCoding":                            {                                "code": "3",                                "display": "Slightly Limited"                            }                        }                    ]                },                {                    "linkId": "questionnaire-kccq-1-3",                    "text": "Walking 100 yards on level ground",                    "answer":                    [                        {                            "valueCoding":                            {                                "code": "0",                                "display": "Extremely Limited"                            }                        }                    ]                },                {                    "linkId": "questionnaire-kccq-1-4",                    "text": "Doing gardening, housework or carrying groceries",                    "answer":                    [                        {                            "valueCoding":                            {                                "code": "4",                                "display": "Not at all Limited"                            }                        }                    ]                },                {                    "linkId": "questionnaire-kccq-1-5",                    "text": "Climbing a flight of stairs without stopping",                    "answer":                    [                        {                            "valueCoding":                            {                                "code": "2",                                "display": "Moderately Limited"                            }                        }                    ]                },                {                    "linkId": "questionnaire-kccq-1-6",                    "text": "Jogging or hurrying (as if to catch a bus)",                    "answer":                    [                        {                            "valueCoding":                            {                                "code": "3",                                "display": "Slightly Limited"                            }                        }                    ]                }            ]        },        {            "linkId": "questionnaire-kccq-section-2",            "item":            [                {                    "linkId": "questionnaire-kccq-2-1",                    "text": "Compared with 2 weeks ago, have your symptoms of heart failure (for example, shortness of breath, fatigue, or ankle swelling) changed? My symptoms of heart failure are now…",                    "answer":                    [                        {                            "valueCoding":                            {                                "code": "0",                                "display": "Much worse"                            }                        }                    ]                },                {                    "linkId": "questionnaire-kccq-2-2",                    "text": "Over the past 2 weeks, how many times have you had swelling in your feet, ankles or legs when you woke up in the morning?",                    "answer":                    [                        {                            "valueCoding":                            {                                "code": "3",                                "display": "Less than once a week"                            }                        }                    ]                },                {                    "linkId": "questionnaire-kccq-2-3",                    "text": "Over the past 2 weeks, how much has swelling in your feet, ankles or legs bothered you?",                    "answer":                    [                        {                            "valueCoding":                            {                                "code": "2",                                "display": "Moderately bothersome"                            }                        }                    ]                },                {                    "linkId": "questionnaire-kccq-2-4",                    "text": "Over the past 2 weeks, on average, how many times has fatigue limited your ability to do what you wanted?",                    "answer":                    [                        {                            "valueCoding":                            {                                "code": "5",                                "display": "Less than once a week"                            }                        }                    ]                },                {                    "linkId": "questionnaire-kccq-2-5",                    "text": "Over the past 2 weeks, how much has your fatigue bothered you?",                    "answer":                    [                        {                            "valueCoding":                            {                                "code": "1",                                "display": "Quite a bit bothersome"                            }                        }                    ]                },                {                    "linkId": "questionnaire-kccq-2-6",                    "text": "Over the past 2 weeks, on average, how many times has shortness of breath limited your ability to do what you wanted?",                    "answer":                    [                        {                            "valueCoding":                            {                                "code": "3",                                "display": "3 or more times a week but not every day"                            }                        }                    ]                },                {                    "linkId": "questionnaire-kccq-2-7",                    "text": "Over the past 2 weeks, how much has your shortness of breath bothered you?",                    "answer":                    [                        {                            "valueCoding":                            {                                "code": "3",                                "display": "Slightly bothersome"                            }                        }                    ]                },                {                    "linkId": "questionnaire-kccq-2-8",                    "text": "Over the past 2 weeks, on average, how many times have you been forced to sleep sitting up in a chair or with at least 3 pillows to prop you up because of shortness of breath?",                    "answer":                    [                        {                            "valueCoding":                            {                                "code": "0",                                "display": "Every night"                            }                        }                    ]                },                {                    "linkId": "questionnaire-kccq-2-9",                    "text": "Heart failure symptoms can worsen for a number of reasons. How sure are you that you know what to do, or whom to call, if your heart failure gets worse?",                    "answer":                    [                        {                            "valueCoding":                            {                                "code": "3",                                "display": "Mostly sure"                            }                        }                    ]                },                {                    "linkId": "questionnaire-kccq-2-10",                    "text": "How well do you understand what things you are able to do to keep your heart failure symptoms from getting worse (for example, regularly weighing yourself, eating a low salt diet etc.)?",                    "answer":                    [                        {                            "valueCoding":                            {                                "code": "0",                                "display": "Do not understand at all"                            }                        }                    ]                },                {                    "linkId": "questionnaire-kccq-2-11",                    "text": "Over the past 2 weeks, how much has your heart failure limited your enjoyment of life?",                    "answer":                    [                        {                            "valueCoding":                            {                                "code": "3",                                "display": "It has slightly limited my enjoyment of life"                            }                        }                    ]                },                {                    "linkId": "questionnaire-kccq-2-12",                    "text": "If you had to spend the rest of your life with your heart failure the way it is right now, how would you feel about this?",                    "answer":                    [                        {                            "valueCoding":                            {                                "code": "0",                                "display": "Completely dissatisfied"                            }                        }                    ]                },                {                    "linkId": "questionnaire-kccq-2-13",                    "text": "Over the past 2 weeks, how often have you felt discouraged or down in the dumps because of your heart failure?",                    "answer":                    [                        {                            "valueCoding":                            {                                "code": "3",                                "display": "I have rarely felt that way"                            }                        }                    ]                }            ]        },        {            "linkId": "questionnaire-kccq-section-3",            "text": "How much does your heart failure affect your lifestyle? Please indicate how your heart failure may have limited your participation in the following activities over the past 2 weeks.",            "item":            [                {                    "linkId": "questionnaire-kccq-3-1",                    "text": "Hobbies,recreational activities",                    "answer":                    [                        {                            "valueCoding":                            {                                "code": "0",                                "display": "Extremely limited"                            }                        }                    ]                },                {                    "linkId": "questionnaire-kccq-3-2",                    "text": "Working or doing household chores",                    "answer":                    [                        {                            "valueCoding":                            {                                "code": "4",                                "display": "Not at all limited"                            }                        }                    ]                },                {                    "linkId": "questionnaire-kccq-3-3",                    "text": "Visiting family or friends",                    "answer":                    [                        {                            "valueCoding":                            {                                "code": "2",                                "display": "Moderately limited"                            }                        }                    ]                },                {                    "linkId": "questionnaire-kccq-3-4",                    "text": "Intimate or sexual relationships",                    "answer":                    [                        {                            "valueCoding":                            {                                "code": "5",                                "display": "Limited for other reasons or did not do the activity"                            }                        }                    ]                }            ]        }    ],    "basedOn":    [        {            "reference": "ServiceRequest/f304953d-314f-4b78-966b-fb81943a314f",            "display": "Kansas City Cardiomyopathy Questionnaire"        }    ]}
