# Supplementary material for: Design, implementation and usability analysis of patient empowerment in ADLIFE project via patient reported outcome measures and shared decision making
Source: BMC Med Inform Decis Mak. 2024 Jun 28;24:185. doi: 10.1186/s12911-024-02588-y (PMC11212241; doi:10.1186/s12911-024-02588-y)
Supplement: Supplementary file 4 — Additional file 4. [file 12911_2024_2588_MOESM4_ESM.rtf]

Additional File 4a.     File format: . rtfb.     Title: An example Score Observation as a FHIR Resourcec.     Description of Data: HL7 FHIR Representation of Score Observation for a KCCQ Questionnaire Response{    "resourceType": "Observation",    "id": "7589c260-ddfd-41cd-992d-ad1ce52df840",    "meta":    {        "source": "http://kroniq.srdc.com.tr/patient",        "versionId": "1",        "lastUpdated": "2023-06-09T19:33:14.404+03:00"    },    "status": "final",    "category":    [        {            "coding":            [                {                    "system": "http://terminology.hl7.org/CodeSystem/observation-category",                    "code": "survey",                    "display": "Survey"                }            ],            "text": "Survey"        }    ],    "subject":    {        "reference": "Patient/6f4cd139-c920-445c-a0e3-e9e60c5ae235"    },    "code":    {        "coding":        [            {                "system": "http://loinc.org",                "code": "86924-8",                "display": "Kansas City Cardiomyopathy Questionnaire"            }        ]    },    "effectiveDateTime": "2023-06-09T16:33:13.876Z",    "component":    [        {            "code":            {                "coding":                [                    {                        "system": "http://kroniq.srdc.com.tr/fhir/CodeSystem/observation-code",                        "code": "kccq-score-physical-limitation",                        "display": "Physical Limitation Score"                    }                ]            },            "valueQuantity":            {                "value": 54.166666666666664,                "system": "http://unitsofmeasure.org",                "code": "{score}"            }        },        {            "code":            {                "coding":                [                    {                        "system": "http://kroniq.srdc.com.tr/fhir/CodeSystem/observation-code",                        "code": "kccq-score-symptom-stability",                        "display": "Symptom Stability Score"                    }                ]            },            "valueQuantity":            {                "value": 25,                "system": "http://unitsofmeasure.org",                "code": "{score}"            }        },        {            "code":            {                "coding":                [                    {                        "system": "http://kroniq.srdc.com.tr/fhir/CodeSystem/observation-code",                        "code": "kccq-score-symptom-frequency",                        "display": "Symptom Frequency Score"                    }                ]            },            "valueQuantity":            {                "value": 52.083333333333336,                "system": "http://unitsofmeasure.org",                "code": "{score}"            }        },        {            "code":            {                "coding":                [                    {                        "system": "http://kroniq.srdc.com.tr/fhir/CodeSystem/observation-code",                        "code": "kccq-score-symptom-burden",                        "display": "Symptom Burden Score"                    }                ]            },            "valueQuantity":            {                "value": 50,                "system": "http://unitsofmeasure.org",                "code": "{score}"            }        },        {            "code":            {                "coding":                [                    {                        "system": "http://kroniq.srdc.com.tr/fhir/CodeSystem/observation-code",                        "code": "kccq-score-total-symptom",                        "display": "Total Symptom Score"                    }                ]            },            "valueQuantity":            {                "value": 51.04166666666667,                "system": "http://unitsofmeasure.org",                "code": "{score}"            }        },        {            "code":            {                "coding":                [                    {                        "system": "http://kroniq.srdc.com.tr/fhir/CodeSystem/observation-code",                        "code": "kccq-score-self-efficacy",                        "display": "Self Efficacy Score"                    }                ]            },            "valueQuantity":            {                "value": 37.5,                "system": "http://unitsofmeasure.org",                "code": "{score}"            }        },        {            "code":            {                "coding":                [                    {                        "system": "http://kroniq.srdc.com.tr/fhir/CodeSystem/observation-code",                        "code": "kccq-score-quality-of-life",                        "display": "Quality of Life Score"                    }                ]            },            "valueQuantity":            {                "value": 50,                "system": "http://unitsofmeasure.org",                "code": "{score}"            }        },        {            "code":            {                "coding":                [                    {                        "system": "http://kroniq.srdc.com.tr/fhir/CodeSystem/observation-code",                        "code": "kccq-score-social-limitation",                        "display": "Social Limitation Score"                    }                ]            },            "valueQuantity":            {                "value": 50,                "system": "http://unitsofmeasure.org",                "code": "{score}"            }        },        {            "code":            {                "coding":                [                    {                        "system": "http://kroniq.srdc.com.tr/fhir/CodeSystem/observation-code",                        "code": "kccq-score-overall-summary",                        "display": "Overall Summary Score"                    }                ]            },            "valueQuantity":            {                "value": 51.302083333333336,                "system": "http://unitsofmeasure.org",                "code": "{score}"            }        },        {            "code":            {                "coding":                [                    {                        "system": "http://kroniq.srdc.com.tr/fhir/CodeSystem/observation-code",                        "code": "kccq-score-clinical-summary",                        "display": "Clinical Summary Score"                    }                ]            },            "valueQuantity":            {                "value": 52.60416666666667,                "system": "http://unitsofmeasure.org",                "code": "{score}"            }        }    ],    "derivedFrom":    [        {            "reference": "QuestionnaireResponse/d0915fc8-823b-4f66-b6b2-6def9271acba"        }    ]}
