# Supplementary material for: Design, implementation and usability analysis of patient empowerment in ADLIFE project via patient reported outcome measures and shared decision making
Source: BMC Med Inform Decis Mak. 2024 Jun 28;24:185. doi: 10.1186/s12911-024-02588-y (PMC11212241; doi:10.1186/s12911-024-02588-y)

Additional File 6

a. File format: pdf

b. Title: The Self-Assessment Questionnaire

c. Description of Data: The Self-Assessment Questionnaire Flow

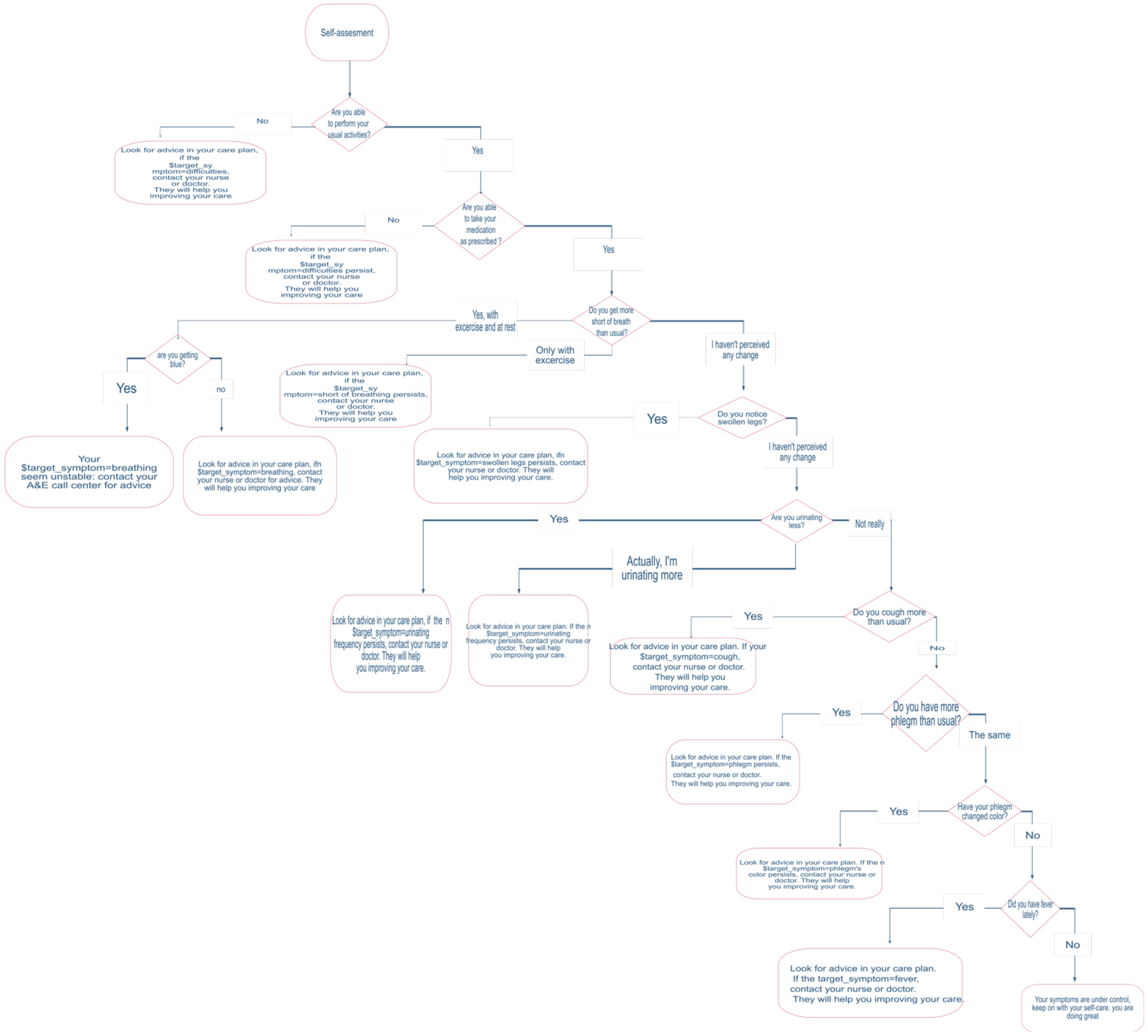

Supplement: Supplementary file 6 — Additional file 6. [file 12911_2024_2588_MOESM6_ESM.pdf]
