# Supplementary material for: Design, implementation and usability analysis of patient empowerment in ADLIFE project via patient reported outcome measures and shared decision making
Source: BMC Med Inform Decis Mak. 2024 Jun 28;24:185. doi: 10.1186/s12911-024-02588-y (PMC11212241; doi:10.1186/s12911-024-02588-y)
Supplement: Supplementary file 7 — Additional file 7. [file 12911_2024_2588_MOESM7_ESM.pdf]

Additional File 7

a. File format: .pdf

b. Title: The 'Shared decision-Making on inhalation medicine in patients of COPD' Decision aid

c. Description of Data: A decision aid that can be assigned to the patient in order to assess which inhalation medication will best suit the patient, asking patient to assess what is most important to them and what matters less

Questions about the patient's preferences

In order to assess which inhalation medication will best suit you, we will ask you to assess what is most important to you and what matters less to you.

Give each of the following questions points according to how important it is for you. You have a total of 10 points you can distribute according to what you consider to be most important to you.

What is most important to you:
(Type a number here. The sum of the three numbers shall give 10 total).

- a) Keeping the daily 'frequency' of inhalation medication intake (number of times you should take inhalation medication daily) as low as possible ☐
- b) Keeping the number of different inhalation device you need to use daily as low as possible? ☐
- c) Keeping the cost of medication as low as possible? ☐
