# Supplementary material for: Design, implementation and usability analysis of patient empowerment in ADLIFE project via patient reported outcome measures and shared decision making
Source: BMC Med Inform Decis Mak. 2024 Jun 28;24:185. doi: 10.1186/s12911-024-02588-y (PMC11212241; doi:10.1186/s12911-024-02588-y)
Supplement: Supplementary file 8 — Additional file 8. [file 12911_2024_2588_MOESM8_ESM.rtf]

Additional File 8a.     File format: .rtfb.     Title: An example Decision Aid as a FHIR Resourcec.     Description of Data: HL7 FHIR Representation of ‘Shared decision-Making on inhalation medicine in patients of COPD’ Decision aid as a Questionnaire Resource instance{    "resourceType": "Questionnaire",    "id": "q-sdmim",    "language": "gb",    "meta":    {        "tag":        [            {                "system": "http://kroniq.srdc.com.tr/fhir/CodeSystem/questionnaire-category",                "code": "patient"            }        ]    },    "title": "Shared decision-Making on inhalation medicine in patients of COPD",    "extension":    [        {            "url": "http://kroniq.srdc.com.tr/fhir/StructureDefinition/optionalQuestionnaire",            "valueReference":            {                "reference": "Questionnaire/q-sdmimprof",                "display": "Shared decision-Making on inhalation medicine in patients of COPD - Professional Questions",                "identifier": {                    "system": "http://kroniq.srdc.com.tr/fhir/CodeSystem/questionnaire-code",                    "value": "sdmimprof"                }            }        },        {            "url": "http://adlifeproject.com/fhir/StructureDefinition/health-outcome-area",            "valueCodeableConcept":            {                "coding":                [                    {                        "system": "http://adlifeproject.com/fhir/CodeSystem/health-outcome-area",                        "code": "healthcare-responsiveness"                    }                ]            }        },        {            "url": "http://adlifeproject.com/fhir/StructureDefinition/health-outcome-dimension",            "valueCodeableConcept":            {                "coding":                [                    {                        "system": "http://adlifeproject.com/fhir/CodeSystem/health-outcome-dimension",                        "code": "continuity-of-care"                    }                ]            }        }    ],    "status": "active",    "subjectType":    [        "Patient"    ],    "date": "2022-11-30T11:00:00+03:00",    "code":    [        {            "system": "http://kroniq.srdc.com.tr/fhir/CodeSystem/questionnaire-code",            "code": "sdmim",            "display": "Shared decision-Making on inhalation medicine in patients of COPD"        }    ],    "item":    [        {            "linkId": "questionnaire-sdmim-section-1",            "type": "group",            "text": "Give each of the following options points according to how important it is for you. You have a total of 10 points you can distribute according to what you consider to be most important to you.",            "extension":            [                {                    "url": "http://hl7.org/fhir/StructureDefinition/maxValue",                    "valueInteger": 10                }            ],            "item":            [                {                    "linkId": "questionnaire-sdmim-1-1",                    "text": "Keeping the daily ’frequency’ of inhalation medication intake (number of times you should take inhalation medication daily) as low as possible",                    "type": "integer",                    "required": true,                    "extension":                    [                        {                            "url": "http://hl7.org/fhir/StructureDefinition/minValue",                            "valueInteger": 0                        },                        {                            "url": "http://hl7.org/fhir/StructureDefinition/maxValue",                            "valueInteger": 10                        },                        {                            "url": "http://hl7.org/fhir/StructureDefinition/questionnaire-sliderStepValue",                            "valueInteger": 1                        }                    ],                    "initial":                    [                        {                            "valueInteger": 0                        }                    ]                },                {                    "linkId": "questionnaire-sdmim-1-2",                    "text": "Keeping the number of different inhalation device you need to use daily as low as possible",                    "type": "integer",                    "required": true,                    "extension":                    [                        {                            "url": "http://hl7.org/fhir/StructureDefinition/minValue",                            "valueInteger": 0                        },                        {                            "url": "http://hl7.org/fhir/StructureDefinition/maxValue",                            "valueInteger": 10                        },                        {                            "url": "http://hl7.org/fhir/StructureDefinition/questionnaire-sliderStepValue",                            "valueInteger": 1                        }                    ],                    "initial":                    [                        {                            "valueInteger": 0                        }                    ]                },                {                    "linkId": "questionnaire-sdmim-1-3",                    "text": "Keeping the cost of medication as low as possible",                    "type": "integer",                    "required": true,                    "extension":                    [                        {                            "url": "http://hl7.org/fhir/StructureDefinition/minValue",                            "valueInteger": 0                        },                        {                            "url": "http://hl7.org/fhir/StructureDefinition/maxValue",                            "valueInteger": 10                        },                        {                            "url": "http://hl7.org/fhir/StructureDefinition/questionnaire-sliderStepValue",                            "valueInteger": 1                        }                    ],                    "initial":                    [                        {                            "valueInteger": 0                        }                    ]                }            ]        }    ]}
