# Supplementary material for: Design, implementation and usability analysis of patient empowerment in ADLIFE project via patient reported outcome measures and shared decision making
Source: BMC Med Inform Decis Mak. 2024 Jun 28;24:185. doi: 10.1186/s12911-024-02588-y (PMC11212241; doi:10.1186/s12911-024-02588-y)
Supplement: Supplementary file 9 — Additional file 9. [file 12911_2024_2588_MOESM9_ESM.pdf]

Additional File 9

a. File format: .pdf

b. Title: QUIS7 Questionnaire for Patients and Informal Caregivers

c. Description of Data: QUIS7 usability questionnaire for patients and their informal caregivers

ADLIFE Usability Testing

Dear participant,

Your feedback is very valuable to us and we appreciate your participation in the demonstration workshop and for working through the usage scenario. In the following 20 minutes we kindly ask you fill in the "QUIS" questionnaire. The QUIS (Questionnaire for User Interaction Satisfaction) is a tool to assess your personal satisfaction with how the ADLIFE software is presented to you.

The questionnaire asks for your feedback on your overall satisfaction with the ADLIFE software. It does that along several topics, including: your overall reaction to the software; the screen; the terminology used; learning factors, the capabilities of the software and how you experienced them, the user manuals that you used. You can save your progress anytime during the survey and resume it later by following the original link to the questionnaire.

Thank you very much in advance.

Please answer all questions by ticking the most appropriate box for each of the following statements:

1. Personal Information

Do you have a chronic condition or do you care for someone who does?

☐ I have a chronic condition ☐ I am a carer of a person who has a chronic condition

If you are a carer of a person with a chronic condition, please answer the questions on behalf of the person you are caring for.

2. Overall Reaction to the ADLIFE System

2.1 I find the ADLIFE system	Terrible 1	2	3	4	5	6	7	8	9 Wonderful	NA
2.2 I find the ADLIFE system	Frustrating 1	2	3	4	5	6	7	8	9 Satisfying	NA
2.3 I find the ADLIFE system	Dull 1	2	3	4	5	6	7	8	9 Stimulating	NA
2.4 I find the ADLIFE system	Difficult 1	2	3	4	5	6	7	8	9 Easy	NA

2.5 I find the ADLIFE system *(Clarification: the system allows all the functions required by the user to be carried out)*	Inadequate Power 1	2	3	4	5	6	7	8	9 Adequate Power	NA
2.6 I find the ADLIFE system	Rigid 1	2	3	4	5	6	7	8	9 Flexible	NA
**3. The Screen**										
3.1 The characters on the computer screen are	Hard to read 1	2	3	4	5	6	7	8	9 Easy to read	NA
3.1.1 The image of characters is	Fuzzy 1	2	3	4	5	6	7	8	9 Sharp	NA
3.2 The highlighting on the screen is	Unhelpful 1	2	3	4	5	6	7	8	9 Helpful	NA
3.2.1 The use of bolding is	Unhelpful 1	2	3	4	5	6	7	8	9 Helpful	NA
3.3 The screen layouts were helpful	Never 1	2	3	4	5	6	7	8	9 Always	NA
3.3.1 The amount of information that can be displayed on screen is	Inadequate 1	2	3	4	5	6	7	8	9 Adequate	NA
3.3.2 The arrangement of information on screen is	Illogical 1	2	3	4	5	6	7	8	9 Logical	NA
3.4 The sequence of screens is	Confusing 1	2	3	4	5	6	7	8	9 Clear	NA
3.4.1 The next screen in a sequence is	Unpredictable 1	2	3	4	5	6	7	8	9 Predictable	NA
3.4.2 Going back to the previous screen is	Impossible 1	2	3	4	5	6	7	8	9 Easy	NA
3.4.3 Progression of tasks is	Confusing 1	2	3	4	5	6	7	8	9 Clearly marked	NA
Please write your comments about the screens here										
**4. Terminology and System Information**										
4.1 The use of terminology throughout system is	Inconsistent 1	2	3	4	5	6	7	8	9 Consistent	NA
4.2 The terminology relates well to the activity I am doing	Never 1	2	3	4	5	6	7	8	9 Always	NA
4.2.1 Computer terminology is used	Too frequently 1	2	3	4	5	6	7	8	9 Appropriately	NA
4.2.2 Terminology on the screen is	Ambiguous 1	2	3	4	5	6	7	8	9 Precise	NA

4.3 Messages that appear on screen are	Inconsistent 1	2	3	4	5	6	7	8	9 Consistent	NA
4.3.1 The position of instructions on the screen is	Inconsistent 1	2	3	4	5	6	7	8	9 Consistent	NA
4.4 Messages that appear on screen are	Confusing 1	2	3	4	5	6	7	8	9 Clear	NA
4.4.1 Instructions for commands or functions are	Confusing 1	2	3	4	5	6	7	8	9 Clear	NA
4.4.2 Instructions for correcting errors are	Confusing 1	2	3	4	5	6	7	8	9 Clear	NA
4.5 The computer keeps me informed about what it is doing	Never 1	2	3	4	5	6	7	8	9 Always	NA
4.5.1 Animated cursors (different mouse pointer shapes) keep me informed	Never 1	2	3	4	5	6	7	8	9 Always	NA
4.5.2 Performing an operation leads to a predictable result	Never 1	2	3	4	5	6	7	8	9 Always	NA
4.5.3 Controlling the amount of feedback is	Impossible 1	2	3	4	5	6	7	8	9 Easy	NA
4.5.4 The length of delay between operations is	Unacceptable 1	2	3	4	5	6	7	8	9 Acceptable	NA
4.6 Error messages are	Unhelpful 1	2	3	4	5	6	7	8	9 Helpful	NA
4.6.1 Error messages clarify the problem	Never 1	2	3	4	5	6	7	8	9 Always	NA
4.6.2 Phrasing of error messages is	Unpleasant 1	2	3	4	5	6	7	8	9 Pleasant	NA
Please write your comments about terminology and system information here										
**5. Learning**										
5.1 Learning to operate the system is	Difficult 1	2	3	4	5	6	7	8	9 Easy	NA
5.1.1 Getting started is	Difficult 1	2	3	4	5	6	7	8	9 Easy	NA
5.1.2 Time to learn to use the system is	Slow 1	2	3	4	5	6	7	8	9 Fast	NA
5.2 Exploration of features by trial and error is	Discouraging 1	2	3	4	5	6	7	8	9 Encouraging	NA

5.3 Remembering menu options and steps in the workflow is	Difficult 1	2	3	4	5	6	7	8	9 Easy	NA
5.4 The number of steps per task is	Too many 1	2	3	4	5	6	7	8	9 Just right	NA
5.5 Feedback on the completion of sequence of steps is	Unclear 1	2	3	4	5	6	7	8	9 Clear	NA
Please write your comments about learning here										
**6. Multimedia**										
6.1 Quality of still pictures/photographs is	Bad 1	2	3	4	5	6	7	8	9 Good	NA
6.1.1 Pictures/Photos are	Fuzzy 1	2	3	4	5	6	7	8	9 Clear	NA
6.1.2 Picture/Photo brightness is	Dim 1	2	3	4	5	6	7	8	9 Bright	NA
6.2 Colours used are	Unnatural 1	2	3	4	5	6	7	8	9 Natural	NA
6.1.2 Amount of colours available is	Inadequate 1	2	3	4	5	6	7	8	9 Adequate	NA
Please write your comments about multimedia here										
**7. Training Material**										
7.1 The training workshop or training video was	Useless 1	2	3	4	5	6	7	8	9 Helpful	NA
7.2 The terminology used in the manual is	Confusing 1	2	3	4	5	6	7	8	9 Clear	NA
7.3 The information from the manual is easily understood	Never 1	2	3	4	5	6	7	8	9 Always	NA
7.4 Finding a solution to a problem using the manual is	Impossible 1	2	3	4	5	6	7	8	9 Easy	NA
7.5 Amount of help given is	Inadequate 1	2	3	4	5	6	7	8	9 Adequate	NA
7.6 Placement of help messages on the screen is	Confusing 1	2	3	4	5	6	7	8	9 Clear	NA
7.7 Accessing help messages is	Difficult 1	2	3	4	5	6	7	8	9 Easy	NA
Please write your comments about training material here										

8. System Capabilities

8.1 System speed is	Too slow 1	2	3	4	5	6	7	8	9 Fast enough	NA
8.1.1 Response time for most operations is	Too slow 1	2	3	4	5	6	7	8	9 Fast enough	NA
8.2 The system is reliable	Never 1	2	3	4	5	6	7	8	9 Always	NA
8.2.1 System failures occur	Frequently 1	2	3	4	5	6	7	8	9 Seldom	NA
8.2.2 The system warns you about potential problems	1 Never	2	3	4	5	6	7	8	9 Always	NA
8.3 Correcting your mistakes is	Difficult 1	2	3	4	5	6	7	8	9 Easy	NA
8.3.1 Correcting typos is	Complex 1	2	3	4	5	6	7	8	9 Simple	NA
8.3.2 Ability to undo operations is	Inadequate 1	2	3	4	5	6	7	8	9 Adequate	NA
8.4 The ease of operation depends on my level of experience	Never 1	2	3	4	5	6	7	8	9 Always	NA
8.4.1 I can accomplish tasks knowing only a few commands	With difficulty 1	2	3	4	5	6	7	8	9 Easily	NA

Please write your comments about system capabilities here

9. Your opinion matters. Please leave either a general or a specific comment below if you would like to. If you are concerned about certain sections, just mention their number as a reference point.

Thank you!
