# Supplementary material for: Design, implementation and usability analysis of patient empowerment in ADLIFE project via patient reported outcome measures and shared decision making
Source: BMC Med Inform Decis Mak. 2024 Jun 28;24:185. doi: 10.1186/s12911-024-02588-y (PMC11212241; doi:10.1186/s12911-024-02588-y)
Supplement: Supplementary file 11 — Additional file 11. [file 12911_2024_2588_MOESM11_ESM.docx]

**Additional File 11**

1. File format: .docx
2. Title: ADLIFE Study Group
3. Description of Data: Members of ADLIFE Study Group Members

**ADLIFE Study Group**

Gokce B. Laleci Erturkmen^1^ Natassia Kamilla Juul^2^, Irati Erreguerena Redondo^3^, Dolores Verdoy Berastegui^3^, Juan De La Torre^3^, Mustafa Yuksel^1^, Bunyamin Sarigul^1^, Gokhan Yilmaz^1^, Sarah N. Lim Choi Keung^4^, Theodoros N. Arvanitis^4^, Thea Damkjaer Syse^2^, Janika Bloemeke^5^, Rachelle Kaye^6^, Anne Dichmann Sorknæs^2^ , Itziar Vergara^7^, Lisa McCann^8^, Margaret Moore^8^, Morven Miller^8^, Marie Holm-Sherman^9^, Mikael Lilja^9^, Harpal Randeva^10^, Ioannis Kyrou^10^, Tim Robbins^10^, Fritz Arndt^11^, Ane Fullaondo-Zabala^3^, Ania Gorostiza^3^, Borja Garcia-Lorenzo^3^, Esteban de Manuel Keenoy^3^, Igor Larrañaga^3^, Maider Mateo-Abad^3^, Nerea González^3^, Oliber Groene^5^, Roma Maguire^8^, John Connaghan^10^, Elinor Dahary Halevy^6^, Baraka Nahir^6^

^1^ SRDC Software Research Development & Consultancy Corp, ODTU Teknokent Silikon Blok Kat:1 No:16 Cankaya/Ankara 06800, Turkey

^2^ Medical Department M/FAM, OUH, Svendborg Hospital, Baagøes Allé 15, 5700 Svendborg, Denmark

^3^ Kronikgune Institute for Health Services Research, Ronda de Azkue 1, Torre del Bilbao Exhibition Centre, 48902 Barakaldo, Basque Country, Spain

^4^ Electronic, Electrical and Systems Engineering, School of Engineering, University of Birmingham, Birmingham, B15 2TT, United Kingdom

^5^ OptiMedis AG, Burchardstrasse 17, 20095 Hamburg, Germany

^6^ Assuta Medical Centers Ltd., Ha-barzel St. 10, Tel Aviv, 69710, Israel

^7^ Biodonostia Health Research Institute, Paseo Dr. Begiristain s/n, 20014 Donostia, Basque Country, Spain

^8^ Department of Computer and Information Sciences, University of Strathclyde, Glasgow. G1 1XQ

^9^ Department of Public Health and Clinical Medicine, Unit of Research, Education and Development Östersund, Umeå University, Umeå, Sweden.

^10^ Digital & Data Driven Research Unit, University Hospitals Coventry & Warwickshire NHS Trust, Clifford Bridge Road, Coventry, CV2 2DX

^11^ Gesunder Werra-Meißner-Kreis GmbH, 37269 Eschwege, Germany
