# Supplementary material for: Design, implementation and usability analysis of patient empowerment in ADLIFE project via patient reported outcome measures and shared decision making
Source: BMC Med Inform Decis Mak. 2024 Jun 28;24:185. doi: 10.1186/s12911-024-02588-y (PMC11212241; doi:10.1186/s12911-024-02588-y)
Supplement: Supplementary file 12 — Additional file 12. [file 12911_2024_2588_MOESM12_ESM.docx]

**Additional File 12**

1. File format: .docx
2. Title: Supplementary File Legends
3. Description of Data: List of supplementary file ledgers

**Supplementary File Ledgers:**

1. **Additional File 1**
2. File format: . rtf
3. Title: Kansas City Cardiomyopathy Questionnaire (KCCQ) FHIR Resource
4. Description of Data: HL7 FHIR Representation of Kansas City Cardiomyopathy Questionnaire (KCCQ) as a Questionnaire Resource instance
5. **Additional File 2**
6. File format: . rtf
7. Title: An example CarePlan FHIR Resource
8. Description of Data: HL7 FHIR Representation of an example care plan that assigns Kansas City Cardiomyopathy Questionnaire (KCCQ) as a patient activity to be performed by the patient.
9. **Additional File 3**
10. File format: . rtf
11. Title: An example QuestionnaireResponse FHIR Resource for Kansas City Cardiomyopathy Questionnaire (KCCQ)
12. Description of Data: HL7 FHIR Representation of an example questionnaire response for Kansas City Cardiomyopathy Questionnaire (KCCQ)
13. **Additional File 4**
14. File format: . rtf
15. Title: An example Score Observation as a FHIR Resource
16. Description of Data: HL7 FHIR Representation of Score Observation for a KCCQ Questionnaire Response
17. **Additional File 5**
18. File format: pdf
19. Title: The COPD Symptom Reporting Questionnaire
20. Description of Data: The COPD Symptom Reposting Questionnaire Flow
21. **Additional File 6**
22. File format: pdf
23. Title: The Self-Assessment Questionnaire
24. Description of Data: The Self-Assessment Questionnaire Flow
25. **Additional File 7**
26. File format: pdf
27. Title: The ‘Shared decision-Making on inhalation medicine in patients of COPD’ Decision aid
28. Description of Data: A decision aid that can be assigned to the patient in order to assess which inhalation medication will best suit the patient, asking patient to assess what is most important to them and what matters less.
29. **Additional File 8**
30. File format: .rtf
31. Title: An example Decision Aid as a FHIR Resource
32. Description of Data: HL7 FHIR Representation of ‘Shared decision-Making on inhalation medicine in patients of COPD’ Decision aid as a Questionnaire Resource instance
33. **Additional File 9**
34. File format: .pdf
35. Title: QUIS7 Questionnaire for Patients and Informal Caregivers
36. Description of Data: QUIS7 usability questionnaire for patients and their informal caregivers
37. **Additional File 10**

a.     File format: .xlsx

b.     Title: ADLIFE_QUIS7_PEP_July2022

c.     Description of Data: QUIS7 usability questionnaire responses for patients and their informal caregivers for the two site groups: Germany+Spain; UK-England

1. **Additional File 11**
2. File format: .docx
3. Title: ADLIFE Study Group
4. Description of Data: Members of ADLIFE Study Group Members
5. **Additional File 12**
6. File format: .docx
7. Title: Supplementary File Legends
8. Description of Data: List of supplementary file ledgers
